# Ionic Covalent Organic Framework-Based Membranes for Selective and Highly Permeable Molecular Sieving

**DOI:** 10.1021/jacs.3c11542

**Published:** 2024-01-17

**Authors:** Xin Liu, Jinrong Wang, Yuxuan Shang, Cafer T. Yavuz, Niveen M. Khashab

**Affiliations:** † Smart Hybrid Materials Laboratory (SHMs), Advanced Membranes and Porous Materials Center, Department of Chemistry, King Abdullah University of Science and Technology (KAUST), Thuwal 23955-6900, Kingdom of Saudi Arabia; ‡ Oxide & Organic Nanomaterials for Energy & Environment Laboratory, Advanced Membranes and Porous Materials Center, Department of Chemistry, King Abdullah University of Science and Technology (KAUST), Thuwal 23955-6900, Kingdom of Saudi Arabia

## Abstract

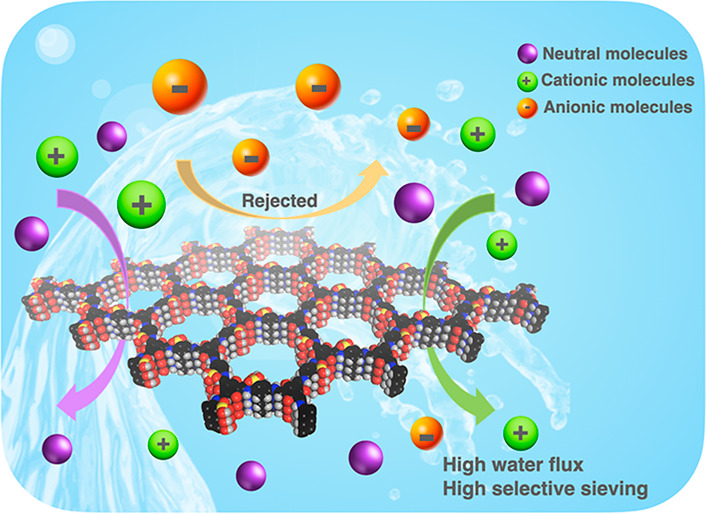

Two-dimensional covalent organic frameworks (COFs) with uniform pores and large surface areas are ideal candidates for constructing advanced molecular sieving membranes. However, a fabrication strategy to synthesize a free-standing COF membrane with a high permselectivity has not been fully explored yet. Herein, we prepared a free-standing TpPa-SO_3_H COF membrane with vertically aligned one-dimensional nanochannels. The introduction of the sulfonic acid groups on the COF membrane provides abundant negative charge sites in its pore wall, which achieve a high water flux and an excellent sieving performance toward water-soluble drugs and dyes with different charges and sizes. Furthermore, the COF membrane exhibited long-term stability, fouling resistance, and recyclability in rejection performance. We envisage that this work provides new insights into the effect of ionic ligands on the design of a broad range of COF membranes for advanced separation applications.

Membrane technology in the context of separation processes has demonstrated an energy-efficient and environmentally friendly potential in contrast to the conventional distillation, condensation, and adsorption approaches.^1−6^ Polymeric and ceramic membranes with varying pore sizes have been developed for molecular sieving with outstanding performance. However, a prevalent issue lies in the lack of continuous uniform and tunable pore channels across these systems, resulting in poor permeability, selectivity, and mechanical properties.^7−16^

Covalent organic frameworks (COFs) are a class of crystalline porous materials that are chemically assembled by molecular building blocks.^17−20^ Reticular chemistry techniques provide various two-dimensional COF structures with large surface areas, ordered and tunable pores, and superior stability, which garner great potential in various fields, such as gas storage,^21,22^ sensing,^23^ energy conversion,^24,25^ and molecular/ion separation.^26,27^ In particular, the inherent long-range ordered structure and permanent porosity make COF-based materials attractive for membrane applications. However, most of the conventional COF synthetic strategies usually result in insoluble and consequently unprocessable microcrystalline powders.^28−33^ Although various approaches to fabricate COF-based mixed matrix membranes have been explored,^34−37^ the resulting membranes usually showed vast internal defects, which limit their practical applications in the membrane industry.^38−40^ To address this drawback, significant efforts have been dedicated to preparing free-standing COF-based membranes.^41−43^ Additionally, the incorporation of ionic modules into COF membranes offers novel functions, particularly in molecular separations with similar molecular weights but different charges. The mass transport within these charged channels is controlled by both the size and charge, thereby providing a unique opportunity to optimize the separation performance.^44−46^

Herein, we present a facile fabrication of a free-standing and highly crystalline sulfonated anionic COF (TpPa-SO_3_H) based membrane with aligned and highly charged one-dimensional (1D) channels for molecular separation in aqueous conditions (Scheme 1). The presence of the sulfonic acid group in the 1D channels significantly increases the membrane permeability, which represents remarkably high water permeance and superior selective sieving for diverse dyes with different sizes and charges.^46,47^ Meanwhile, the recycling experiments and long-term filtration process demonstrated good recyclability, fouling resistance, and superior stability of the TpPa-SO_3_H membrane.^43^

**Scheme 1:**
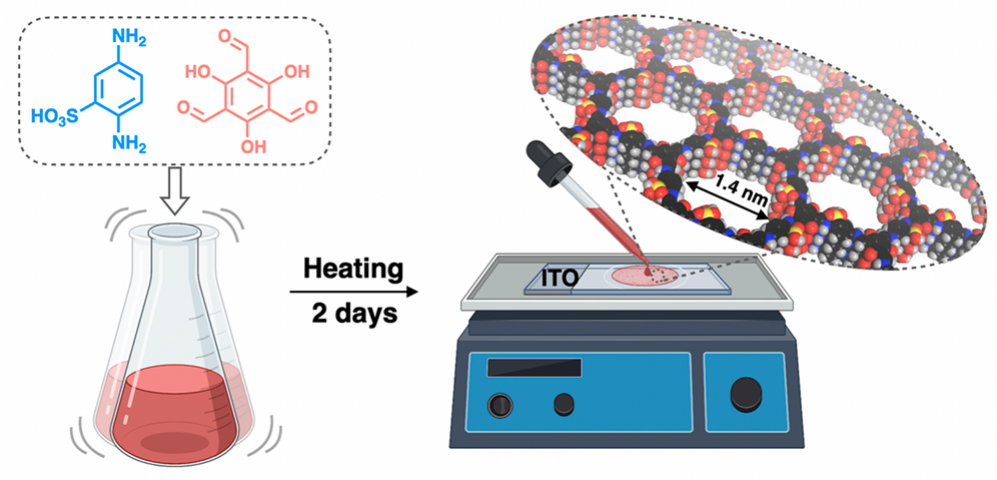
Illustration of the Fabrication Process of the TpPa-SO_3_H Membrane

The COF membrane was successfully synthesized via a Schiff-base condensation reaction on an ITO substrate in a dilute precursor solution comprising 1,3,5-Triformylphloroglucinol (Tp) and 2,5-diaminobenzenesulfonic acid (Pa-SO_3_H) in *N*-methyl pyrrolidone and dimethyl sulfoxide solution (Scheme 1). After condensation for 48 h in an open environment, the ITO substrate was removed to achieve the free-standing TpPa-SO_3_H membrane which was then transferred to a polyacrylonitrile (PAN) support for further characterization (Figures 1a,b, S1, and S2). Using diluted concentrations and shorter reaction time provided much thinner membranes with high water flux compared to reported membranes.^46^ Top-view and cross-sectional scanning electron microscopy (SEM) images revealed that the TpPa-SO_3_H membrane is intact and continuous (Figure 1c–e). Meanwhile, atomic force microscopy (AFM) showed a smooth surface of the membrane with a surface roughness of approximately 0.92 nm (Figure S3). The thickness of the TpPa-SO_3_H membrane was tunable from ∼600 nm to 8.7 μm by altering the concentration of the initial precursors (Figure 1f, Figure S4, and Table S1).

**Figure 1:**
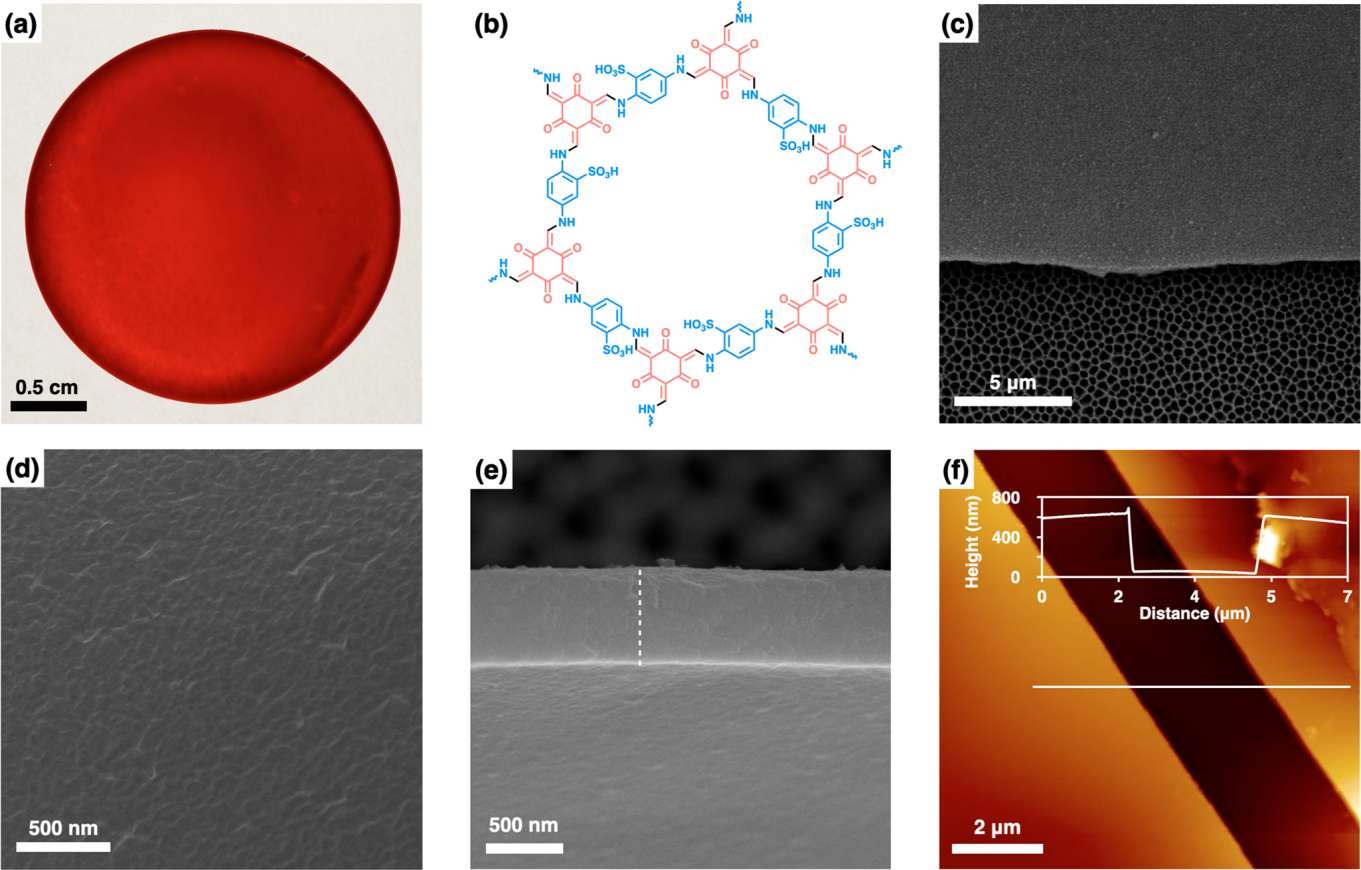
(a) Photograph of the TpPa-SO_3_H membrane. (b) Crystal structure of TpPa-SO_3_H. (c and d) Surface and (e) cross-sectional SEM images of the TpPa-SO_3_H membrane on anodic aluminum oxide (AAO) support. (f) AFM image of TpPa-SO_3_H membrane on mica support and the corresponding thickness profile (along the white line).

The chemical structure of the TpPa-SO_3_H membrane was investigated by Fourier transform infrared (FT-IR) spectroscopy (Figure 2a). The new peaks at 1556 and 1180 cm^–1^ are assigned to C=C and C–N stretching bands, indicating complete enol-to-keto tautomerization. The disappearance of the −C=O (1634 cm^–1^) and −N–H (3334–3425 cm^–1^) stretching vibrations from Tp and Pa-SO_3_H, respectively, suggests a successful formation of the TpPa-SO_3_H. Besides, peaks at 1021 and 1076 cm^–1^ correspond to the O=S=O stretching bands. The condensation was further verified through the ^13^C cross-polarization magic-angle spinning nuclear magnetic resonance (CP/MAS NMR) spectrum. As shown in Figure 2b, the peaks at 182.67, 147.23, and 106.57 ppm validate the successful synthesis of TpPa-SO_3_H.^45^ Furthermore, X-ray photoelectron spectroscopy (XPS) was used to investigate the formation of the β-ketoenamine linkage in the COF structure, which was confirmed by the presence of C 1s, N 1s, O 1s, and S 2p signals (Figure 2c and Figure S5).^48^

**Figure 2:**
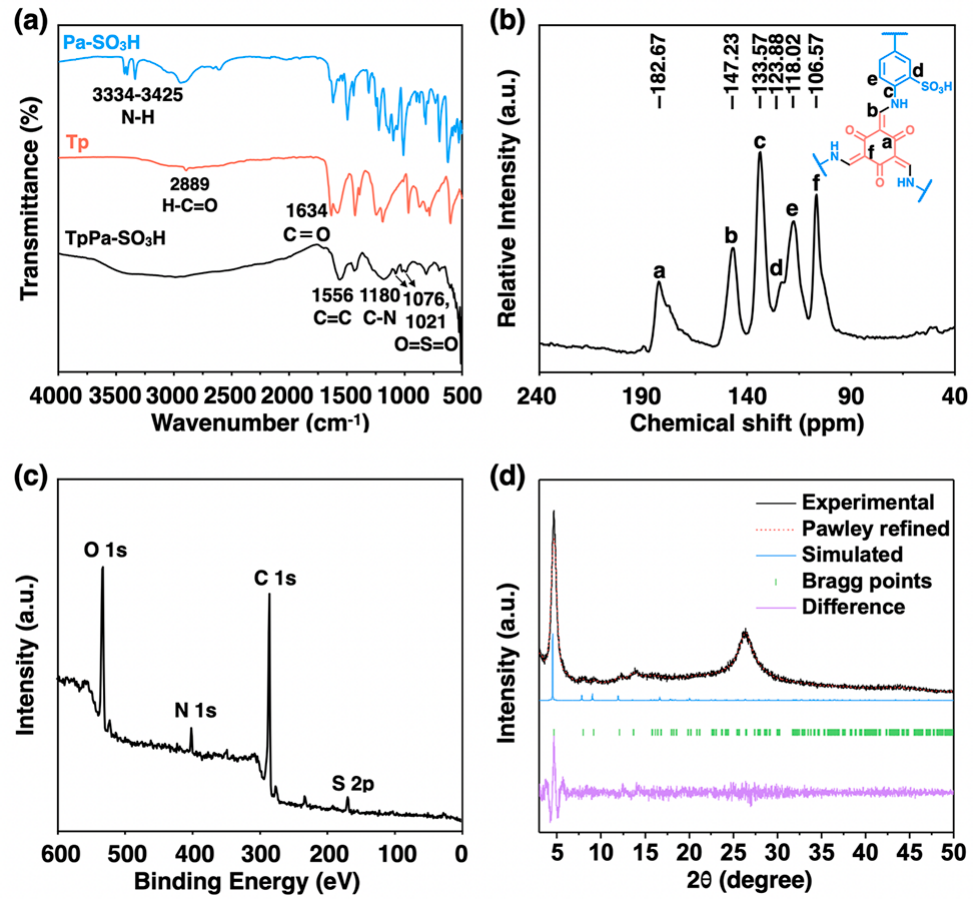
(a) FTIR spectra of the monomers and TpPa-SO_3_H membrane. (b) ^13^C NMR spectrum of TpPa-SO_3_H membrane. (c) XPS survey spectrum of the TpPa-SO_3_H membrane. (d) Comparison of the experimental PXRD patterns of TpPa-SO_3_H membranes with simulated eclipsed stacking model and their Pawley refinement difference (*R*_p_ = 5.18%, *R*_wp_ = 6.63%).

The COF membrane crystallinity was determined by powder X-ray diffraction (PXRD) analysis (Figure 2d and Table S2). The peaks at 4.7° and ∼26.7° correspond to the reflection from the (100) plane and the fine structure of the membrane.^45^ The experimental PXRD patterns match well with the simulated pattern in Figure S6. Different layers were highlighted in different colors, which clearly showed that the layers were stacked in eclipsed 2D stacking mode along the *c*-axis. The Brunauer–Emmett–Teller (BET) surface area of TpPa-SO_3_H calculated from the N_2_ adsorption–desorption isotherm at 77 K was 56.83 m^2^ g^–1^. The pore size distributions revealed that TpPa-SO_3_H has a uniform pore size of 1.42 nm (Figure S7).^45^

To investigate the wettability and chargeability of the TpPa-SO_3_H membrane, water contact angle (WCA) and zeta potential measurements were performed. The water contact angle of TpPa-SO_3_H is 41.29 ± 0.66°, indicating a highly hydrophilic nature due to the sulfonic acid group (Figure S8). As for chargeability, the zeta potential of TpPa-SO_3_H membranes at different pH exhibited a strong negatively charged material (Figure S9). The mechanical strength of the TpPa-SO_3_H membranes was further measured, demonstrating high flexibility and good mechanical strength to withstand pressure-driven filtration (Figure S10). Even after drying, the TpPa-SO_3_H membrane still maintains its flexibility and integrity (Figure S2).

Considering the hydrophilicity and negatively charged nature of the membrane, it was tested for the selective sieving of water-soluble dyes. The pure water flux performance of the COF membrane was initially evaluated, and it was found to exhibit a high water flux of 85.7 LMH. Moreover, studies were also conducted on membranes with different thicknesses (Figure S11). The permeability of pure water is decreased gradually along with an increase in the membrane thickness. However, the permeability of the COF-1 membrane was found to be much higher than that of other reported membranes (Table S3).

To gain insight into the rejection performance of environmentally and industrially relevant molecules, we selected several target dye and drug molecules with different charges and sizes (Figure S12 and Tables S4 and S5). The rejection performance of the COF-1 membranes was evaluated (Figure 3a). For anionic dyes, the membrane can reject Congo red (CR, *M*_w_ = 696.66 Da; 2.56 nm × 0.73 nm), Eriochrome black T (EBT, *M*_w_ = 461.38 Da; 1.55 nm × 0.88 nm), and Fluorescein sodium salt (FSs, *M*_w_ = 376.27 Da; 1.03 nm × 0.96 nm) with a rejection of 99.4%, 98.7%, and 92.2%, respectively. For neutral dyes, the membrane can reject Calcein (CA, *M*_w_ = 622.53 Da; 1.76 nm × 0.88 nm), Rhodamine B base (RBb, *M*_w_ = 42.55 Da; 1.49 nm × 1.15 nm), and p-Nitroaniline (NA, *M*_w_ = 138.12; 0.69 nm × 0.43 nm) with a rejection of 89.3%, 36.1%, and 15.3%, respectively. For cationic dyes, the membrane can reject Alcian blue 8GX (AB, *M*_w_ = 1298.86 Da; 2.22 nm × 2.08 nm), Methylene blue (MB, *M*_w_ = 319.85 Da; 1.52 nm × 0.75 nm), and Crystal violet (CV, *M*_w_ = 407.99 Da; 0.91 nm × 0.91 nm) with a rejection of 93.9%, 82.6%, and 82.6%, respectively. The three colorless filtrates of anionic dyes observed after filtration demonstrated a highly promising anionic dye rejection behavior, as revealed by the significantly decreased concentrations of the permeate (Figure S13). The three neutral dyes showed a smaller decrease in concentration after sieving while still retaining their partial color (Figure S14). For three cationic dyes, a clear color decline was also observed in the filtrates and a clear concentration decline was obtained from UV spectra (Figure S15). To further evaluate the rejection performance, salt rejection behavior of TpPa-SO_3_H membranes was also investigated (Figure S16). The COF membrane displayed an impressive rejection of Na_2_SO_4_ (88.2%) compared to MgSO_4_ (20.6%) and CaCl_2_ (2.4%) due to the negatively charged surface, which repels multivalent anions while attracting multivalent cations. Following the individual rejection tests, mixed dye solutions were used to evaluate the selective molecular separation of the TpPa-SO_3_H membranes (Figure S17). The selective removal of CR from the CR/NA mixture can be observed in the digital photos and UV spectra before and after the membrane filtration.

**Figure 3:**
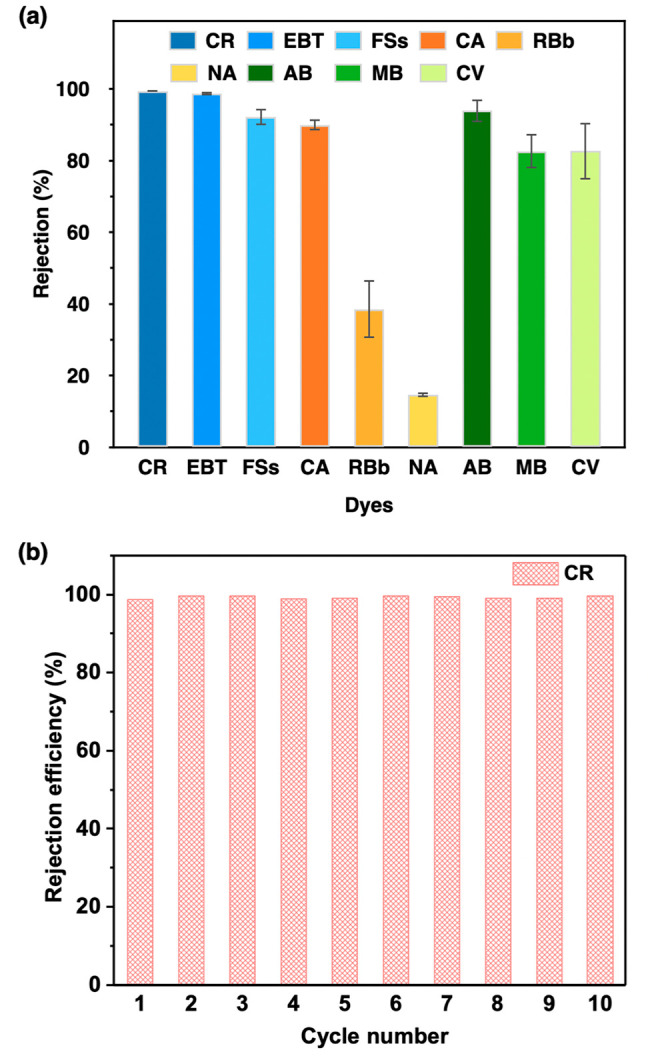
(a) Rejection performance of the TpPa-SO_3_H membrane for Congo red (CR), Eriochrome black T (EBT), Fluorescein sodium salt (FSs), Calcein (CA), Rhodamine B base (RBb), p-Nitroaniline (NA), Alcian blue 8GX (AB), Methylene blue (MB), and Crystal violet (CV) dyes. (b) Cycle performance of CR rejection through the TpPa-SO_3_H membrane.

As a promising separation membrane, recyclability is an essential factor that must be considered for practical applications. Therefore, cycling experiments of TpPa-SO_3_H membranes for different dyes were conducted (Figure 3b and Figure S18). TpPa-SO_3_H membranes displayed an impressive dye rejection efficiency, even after 10 cycles. Moreover, the rejection behavior with different concentrations of dyes was executed. The rejection values were still very high even toward a high concentration of dyes (Figure S19). To highlight the outstanding performance of the membrane, we compared the dye rejection of the TpPa-SO_3_H membrane to those reported in the literature. As shown in Figure 4, the dye rejection performance and pure water flux of the TpPa-SO_3_H membrane are better than the reported values (Table S3).

**Figure 4:**
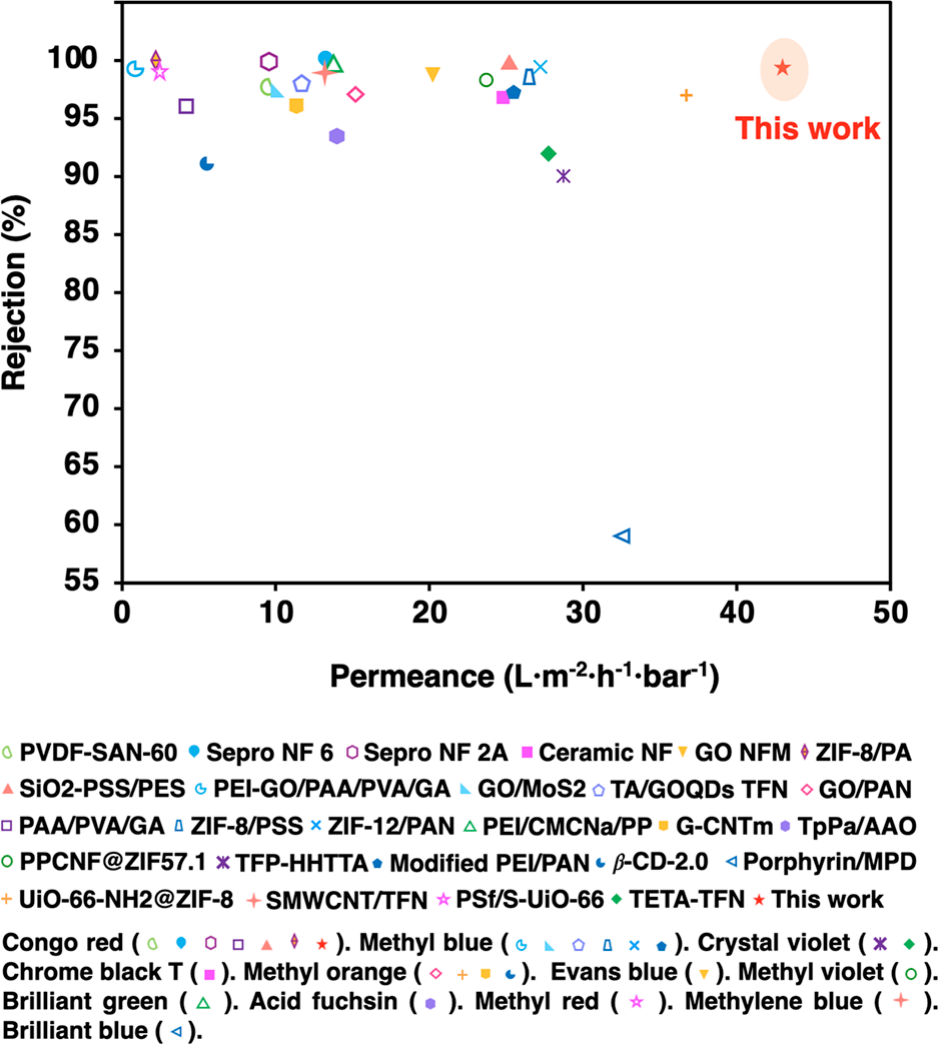
Dye separation performance of the TpPa-SO_3_H membrane in this work and other membranes in the literature.

The stability and fouling resistance of the COF membrane were comprehensively evaluated. The TpPa-SO_3_H membrane exhibited decent antifouling properties and stable rejection performance during a long-term filtration process of dye molecules for more than 30 h (Figure S20). The SEM image of the tested membrane still showed a continuous and uniform surface (Figure S21).^49^ The thermal stability of the TpPa-SO_3_H membrane was examined by thermogravimetric analysis (TGA), which revealed that the membrane was stable up to 250 °C (Figure S22).

The above experimental results prove that the TpPa-SO_3_H membrane had excellent sieving performance for various dye molecules compared to the published literature (Figure 4). To further understand the mechanism of separation behavior, density functional theory (DFT) calculations of binding energy (*E*_be_) between dye molecules and membranes were conducted (Figure S23).^50^ Theoretically, the observed molecular separation is attributed to a combination of “size exclusion” and “electrostatic repulsion”.^51,52^ According to the simulation result, the *E*_be_ value between a cationic molecule (MB) and membranes is the lowest, indicating an electrostatic interaction exists between membranes and cationic molecules. Conversely, the *E*_be_ value between an anionic molecule (EBT) and membranes is much higher than those of the other two combinations, which indicates that there is an electrostatic repulsion interaction between membranes and anionic molecules. Despite the smaller size of Fluorescein sodium salt,^45^ the COF membrane achieved a high rejection value of 92.2%. While for cationic dyes, the rejection efficiency was affected by both electrostatic interaction and molecular size (Figures S24 and S25).^44^ To extend the applicability of separations based on COF membranes, an effective removal of drugs from aqueous solution was also executed (Figure S26 and Table S5). The TpPa-SO_3_H membrane displayed high rejections to drugs with molecular sizes larger than the pore size (Figure S27).^53,54^ These results demonstrated that ionic COF membranes have the potential to be successfully employed for water purification, especially in the pharmaceutical industry.

In summary, we have successfully developed a continuous, free-standing, and flexible anionic COF membrane with aligned one-dimensional channels. The TpPa-SO_3_H membrane exhibited a high water flux toward industrially related dye and drug molecules with different charges and sizes. Moreover, the TpPa-SO_3_H membrane displayed remarkable stability and decent fouling resistance, making it highly promising for micropollutant removal from wastewater treatment and drug purification in the pharmaceutical industry. With its facile preparation strategy, long-term stability, tunable thickness, and superior separation performance, this generation of ionic COF membranes will provide great opportunities for advancing charge-dependent molecular sieving and sustainable separation processes in the future.

## Supporting Information Available

General materials; COF membrane synthetic procedures; computational simulation details; SEM images; AFM images; N_2_ adsorption; mechanical strength measurement; XPS analysis; water contact angle measurement; zeta potential; water flux measurement; dye, salt, and drug rejection measurement; TGA measurement (PDF)
