# Supplementary material for: Ionic Covalent Organic Framework-Based Membranes for Selective and Highly Permeable Molecular Sieving
Source: J Am Chem Soc. 2024 Jan 17;146(4):2313–8. doi: 10.1021/jacs.3c11542 (PMC10835733; doi:10.1021/jacs.3c11542)
Supplement: Supplementary file 1 — ja3c11542_si_001.pdf [file ja3c11542_si_001.pdf]

Supporting Information

Ionic Covalent Organic Frameworks Based Membranes for Selective and Highly Permeable Molecular Sieving

Xin Liu,[a] Jinrong Wang,[a] Yuxuan Shang,[b] Cafer T. Yavuz,[b] Niveen M. Khashab*[a]

[a] Smart Hybrid Materials Laboratory (SHMs), Advanced Membranes and Porous Materials Center, Department of Chemistry, King Abdullah University of Science and Technology (KAUST), Thuwal 23955-6900, Kingdom of Saudi Arabia.

[b] Oxide & Organic Nanomaterials for Energy & Environment Laboratory, Advanced Membranes and Porous Materials Center, Department of Chemistry, King Abdullah University of Science and Technology (KAUST), Thuwal 23955-6900, Kingdom of Saudi Arabia.

*E-mail: niveen.khashab@kaust.edu.sa

Table of Contents

1. Materials and reagents..S3

2. Instruments and characterization S3

3. Methods. S4

Preparation of TpPa-SO_3H membrane. S4

Permeation and rejection performance measurement...S4

4. Figures....S7

5. Tables. S25

6. Supplemental references...S30

1. Materials and reagents

All chemical reagents and solvents from commercial sources were received and used without further purification. Ultrapure water was processed using a Millipore purification system (ELGA PURELAB) with a minimum resistivity of 18.2 MΩ·cm. 1,3,5-Triformylphloroglucinol (Tp) was purchased from Jilin Chinese Academy of Sciences-Yanshen Technology Co., Ltd (Jilin, China). 2,5-Diaminobenzenesulfonic acid (Pa-SO_3H) was purchased from TCI. *p*-Phenylenediamine (PDA), Dimethyl sulfoxide (DMSO), N-methylpyrrolidone (NMP), Congo red, Eriochrome black T, Fluorescein sodium salt, Calcein, Rhodamine B base, p-Nitroaniline, Alcian blue 8GX, Methylene blue, and Crystal violet were obtained from Sigma. Polyacrylonitrile support (PAN, MWCO = 100 kDa) was purchased from Lanjing Membrane Technology Co., Ltd (Shandong, China).

2. Instruments and characterization

The Fourier transform infrared spectroscopy spectrophotometer (FT-IR, Nicolet iS 10) was utilized to analyze the chemical structure of monomers and the Tp-PaSO_3H membrane with a scanning wavelength in the range of 4000-400 cm^{-1} in the attenuated total reflection mode. The solid-state ^{13}C cross-polarisation (CP) magnetic angle spinning (MAS) spectra were collected using a Bruker 400M WB NMR spectrometer. The X-ray photoelectron spectroscopy (XPS) analyses were performed on the Kartos Amicus system. The powder X-ray diffraction (PXRD) patterns were recorded on a D8 ADVANCE diffractometer with Cu Kα radiation (λ = 1.5406 Å) at 40 kV, 40 mA power from 3° to 50° (2θ) with a step size of 0.02° and a scan rate of 5° min^{-1}. The surface and cross-sectional morphologies of the Tp-PaSO_3H membranes were observed via scanning electron microscopy (SEM) using Magellan XHR and Teneo scanning electron microscope. Prior to the measurements, the Tp-PaSO_3H membranes were fixed on the sample stub and coated with a 5 nm-thick iridium layer using a Quorum Q150T sputter coater. The surface roughness and membrane height were conducted by atomic force microscopy (AFM) on a Bruker Dimension Icon at room temperature. The N_2 sorption isotherm at 77 K was collected by using Micromeritics ASAP 2420 surface area and pore size analyzer. The COF membrane sample was degassed at 150 °C for 12 h under vacuum before the measurement. A drop-shape analyzer (Kruss, DSA100, Germany) was employed to measure the water contact angle (WCA) of the membranes.

The filtration performance was measured by a double-beam ultraviolet-visible spectrometer (Shimadzu UV 2600) using quartz cuvettes.

3. Methods.

Preparation of TpPa-SO_3H membrane.

Tp (10 mM) and Pa-SO_3H (13 mM) were dissolved in an NMP and DMSO mixture. The resultant suspension was sonicated for 15 min to form a homogenous solution. Then the solution was directly drop-casting into a clean ITO glass slide in the oven. The reaction was conducted at 60 ℃ for 2 days, yielding a uniform TpPa-SO_3H membrane on the surface of the ITO substrate. The TpPa-SO_3H membrane attached to ITO was washed with acetone, ethanol, and tetrahydrofuran three consecutive times to remove residual monomer fragments. Then it was put into water, the scaled free-standing membrane was peeled off from the ITO substrate. The construction of TpPa membrane followed the same procedure. The thickness of the TpPa-SO_3H membrane could be controlled by altering the initial Tp and Pa-SO_3H concentrations.

Permeation and rejection performance measurement

The filtration performance of the membranes was evaluated using a dead-end mode stirred cell filtration device with an effective permeate area of 3.14 cm^2. The membranes were used directly for permeation and rejection studies with no special activation, in contrast to regular polymer membranes.[3] For each experiment, the stirring speed was set at 300 r.p.m and the flux was collected after 30 min when the flux had reached a steady state.

The pure water flux (Jw) in L m^{-2} h^{-1} was measured at 0.2 Mpa and was calculated according to the following equation:

$$J_w = \frac{V}{A\Delta t} \qquad (1)$$

Where V (L) is the volume of pure water collected in the permeate side during a certain period of Δt (h), and A (m^2) is the effective membrane area, which is approximately 3.14×10^{-4} m^2.

The dye rejection performance of the membranes was measured in 20 ppm, 100 ppm and 300 ppm solutions. The rejection efficiency (R) was measured according to the following equation:

$$R = \left(1 - \frac{C_P}{C_F}\right) \times 100\%. \quad (2)$$

Where R is solute rejection efficiency, and C_P and C_F are the concentration of dyes in the permeate and feed solutions, respectively. The concentration of feed solution, permeate solution, and the retention of dyes was determined with a double-beam ultraviolet-visible spectrometer using quartz cuvettes. The accuracy and reliability of the results were confirmed by testing three membrane samples and averaging the values obtained.

In salt rejection tests, 1000 ppm salts were used for testing rejection behavior of COF membranes. The concentration of feed solution, permeate solution, and retention solution was detected by inductively coupled plasma optical emission spectrometry (ICP-OES, Agilent 5110).

A cycling experiment of the TpPa-SO_3H membrane for Congo red (CR), Eriochrome black T (EBT), Calcein (CA), Alcian blue 8GX (AB), and Methylene blue (MB) interception was conducted, separately. After the first interception for 1h, the membrane was rinsed thoroughly with an aqueous solution for another 1h at ambient conditions. Subsequently, the filtration experiment was repeated several times with the same membrane, using identical conditions. Finally, permeate and feed solutions were collected and analyzed using a UV-vis spectrophotometer.

DFT simulation

The DFT (Density functional theory) calculations based on PBE were conducted using $DMol^3$ package of Material Studio. The binding energy (E_{be}) between the membrane and molecules is expressed by the following equation:

$$Mem(Membrane) + Dye \Leftrightarrow Dye@Mem\ complex$$

The binding energy E_{be} was calculated by DFT calculation results using the following equation:

$$E_{be} = E_{Dye@Mem\ complex} - E_{Mem} - E_{Dye} - BSSE$$

In the above equation, the four terms on the right are respectively the total energy of the Dye@Mem complex, the isolated membrane, and molecule, and the basis set superposition error (BSSE).

4. Figures.

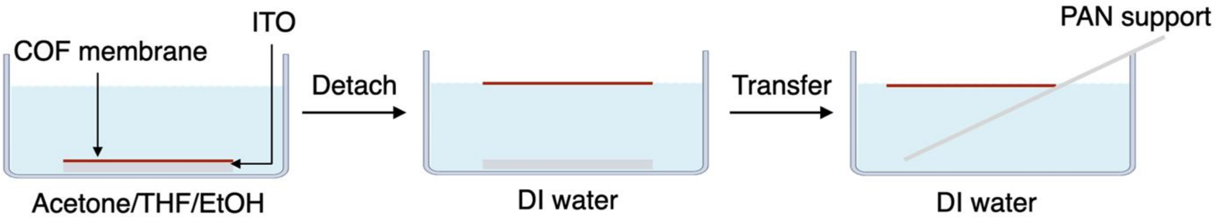

Figure S1. Schematic illustration of the TpPa-SO_3H membrane transfer process.

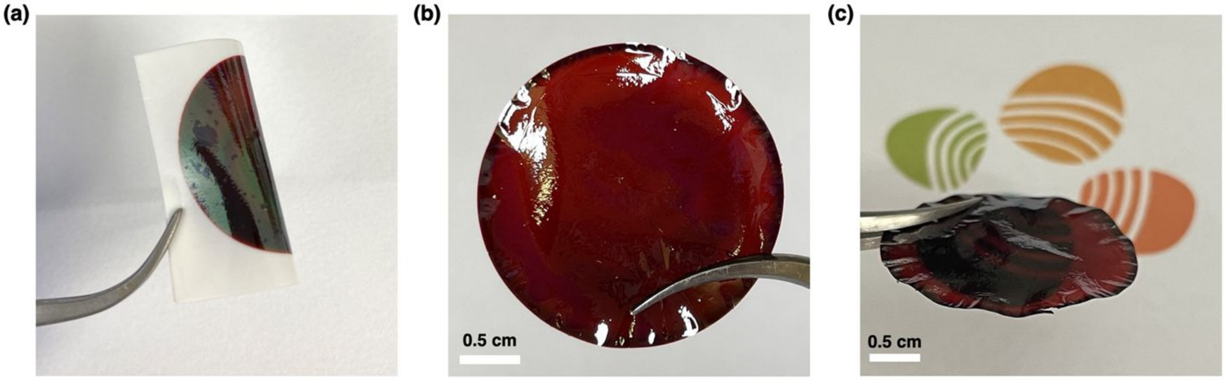

Figure S2. (a) Bent TpPa-SO_3H membrane on PAN substrate. (b) and (c) digital photographs of dry TpPa-SO_3H membrane.

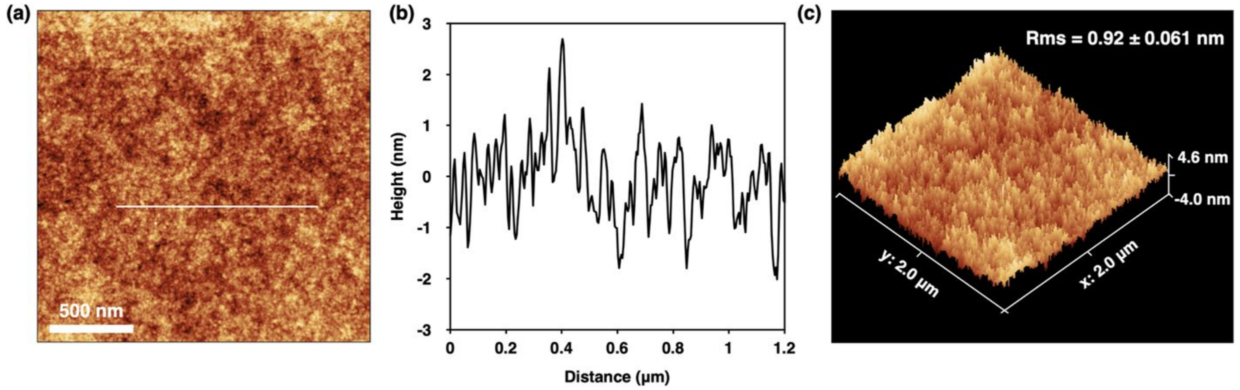

Figure S3. (a) and (b) AFM images of the TpPa-SO_3H membrane and corresponding thickness profile along the white line. (c) 3D AFM images of the TpPa-SO_3H membrane and root-mean-square roughness (Rms).

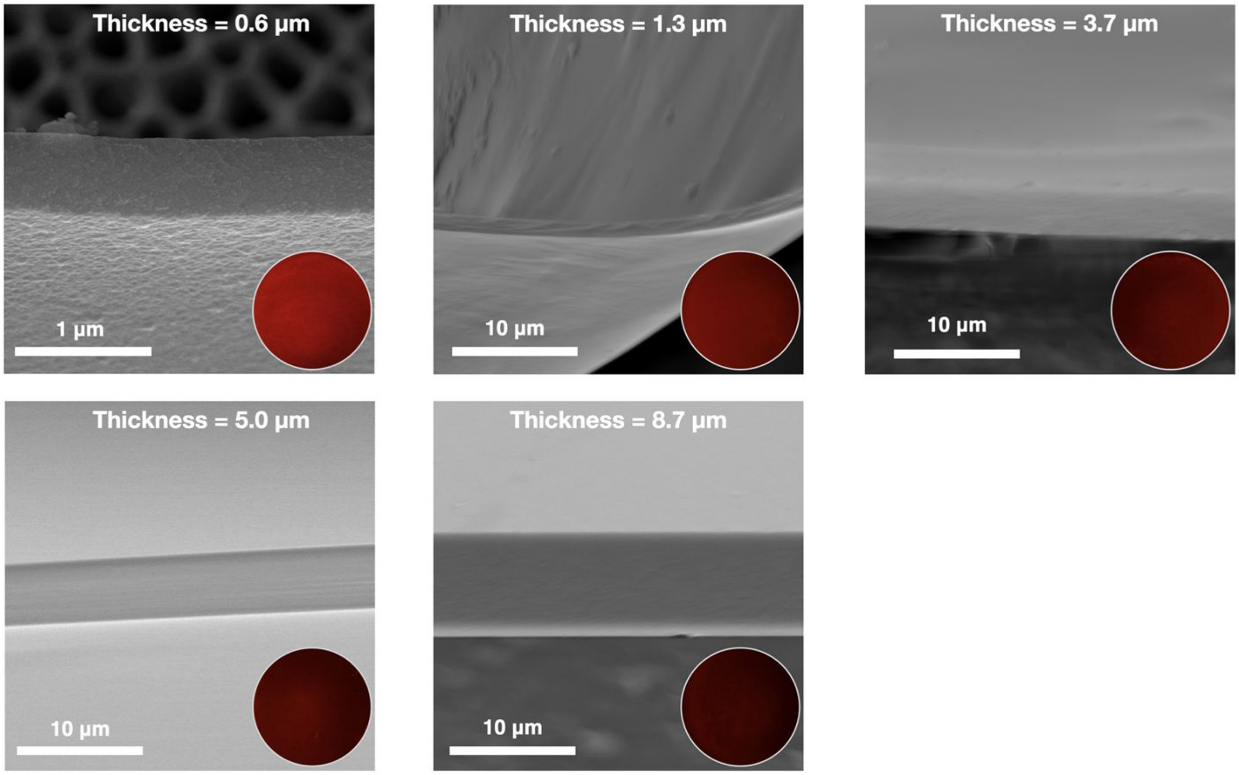

Figure S4. SEM images of free-standing TpPa-SO_3H membranes with different thicknesses. Inset: digital photos of free-standing TpPa-SO_3H membranes.

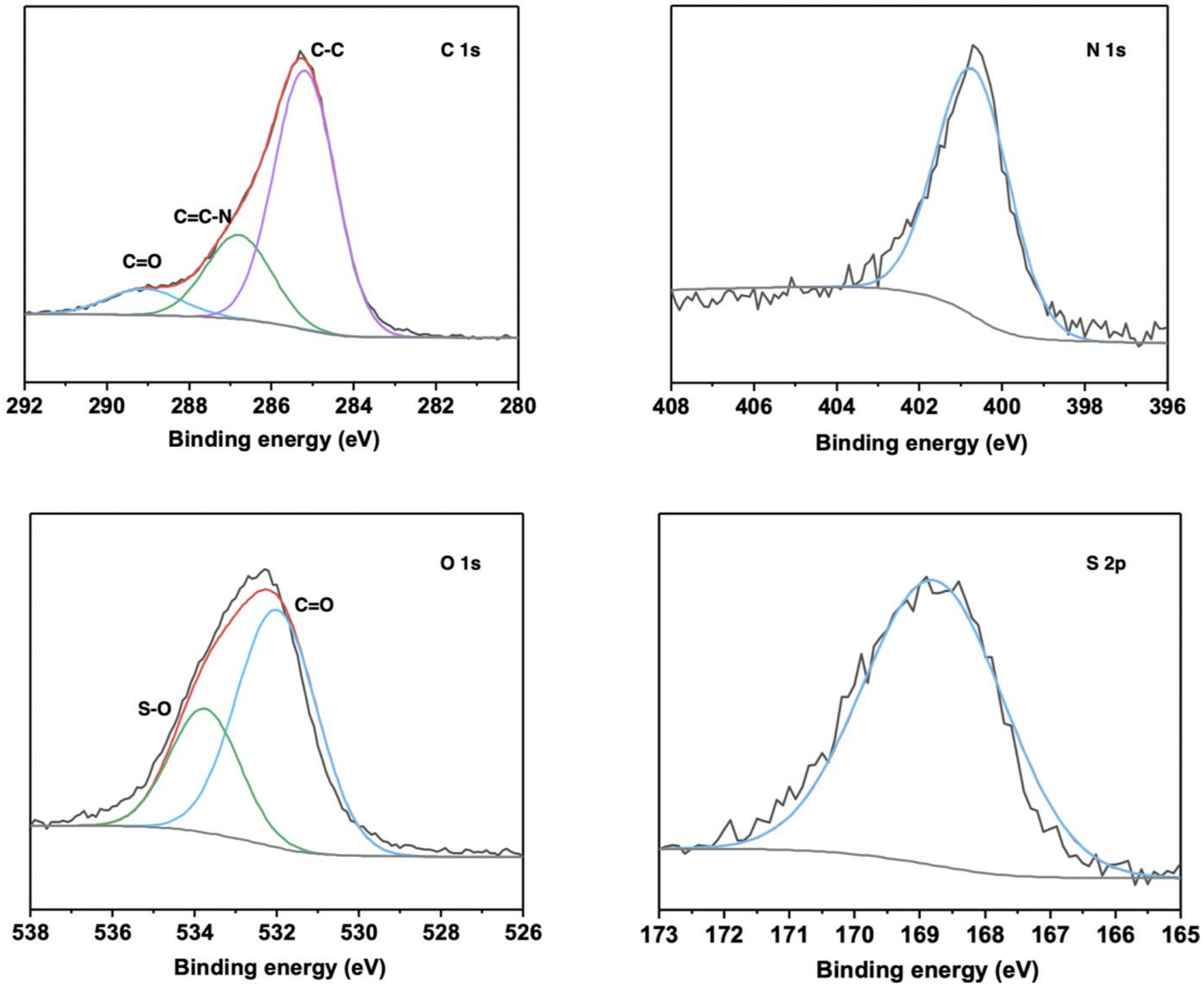

Figure S5. High-resolution XPS spectra of C 1s, N 1s, O 1s, and S 2p.

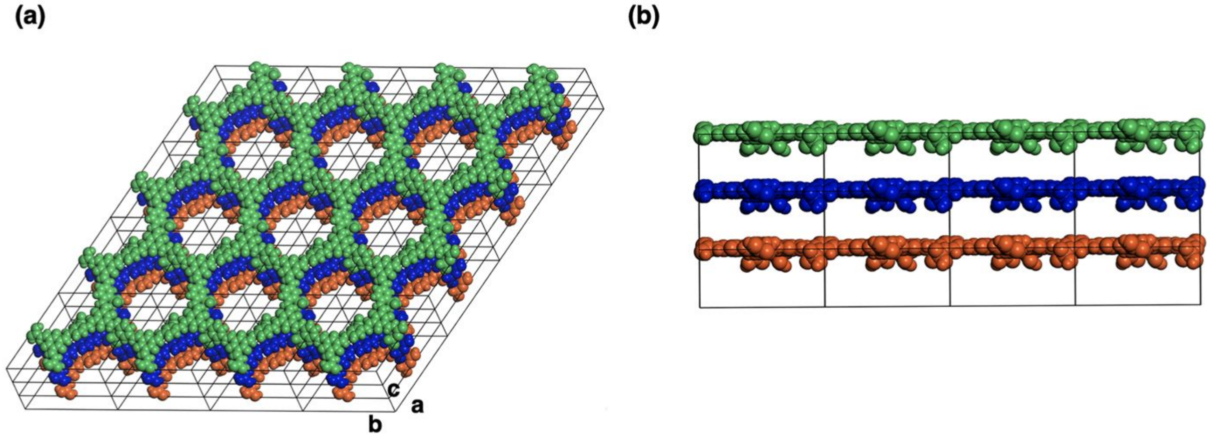

Figure S6. (a) Top view of the simulated TpPa-SO_3H structure. (b) Side view of the simulated TpPa-SO_3H structure showing the inclined eclipsed stacking structure.

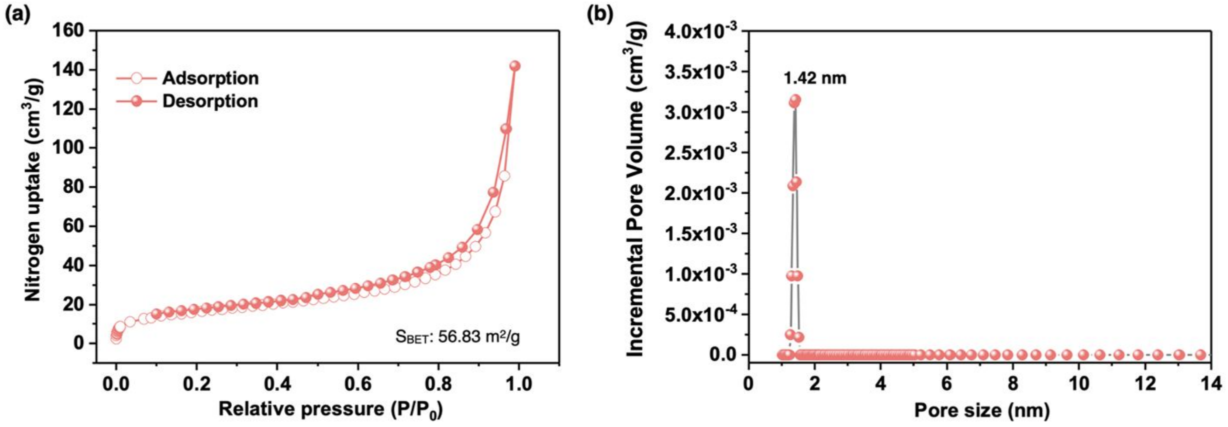

Figure S7. (a) N_2 adsorption-desorption isotherms of TpPa-SO_3H membranes measured at 77 K. (b) The pore size distribution profile was calculated by using the NLDFT model.

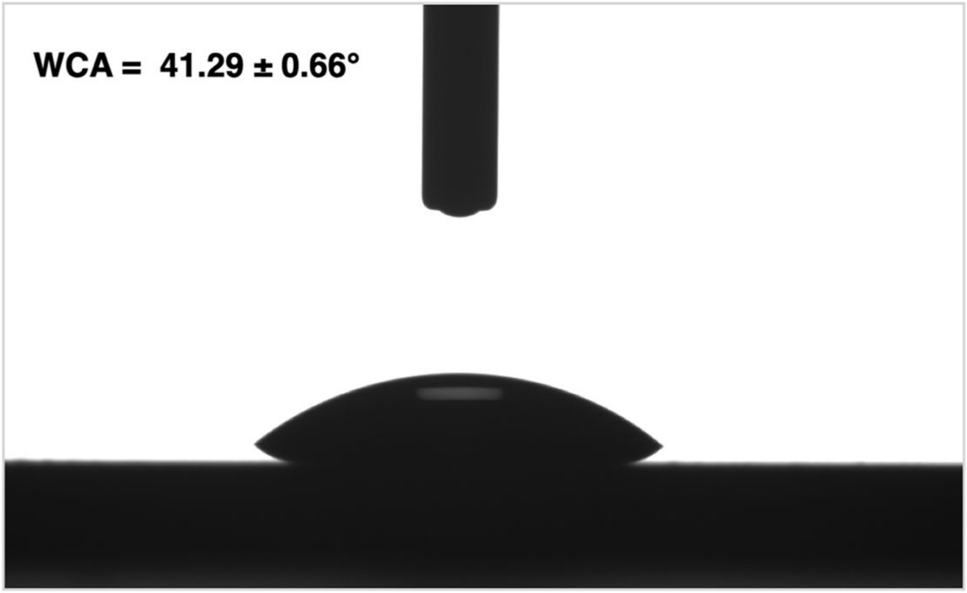

Figure S8. Water contact angle (WCA) image of TpPa-SO_3H membrane at ambient condition.

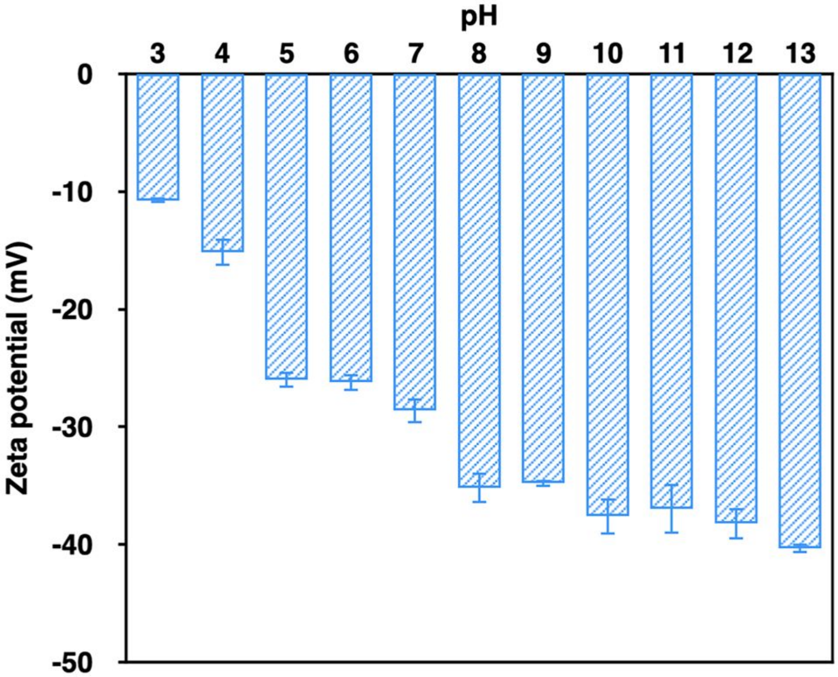

Figure S9. Zeta potential of TpPa-SO_3H membranes at different pH conditions.

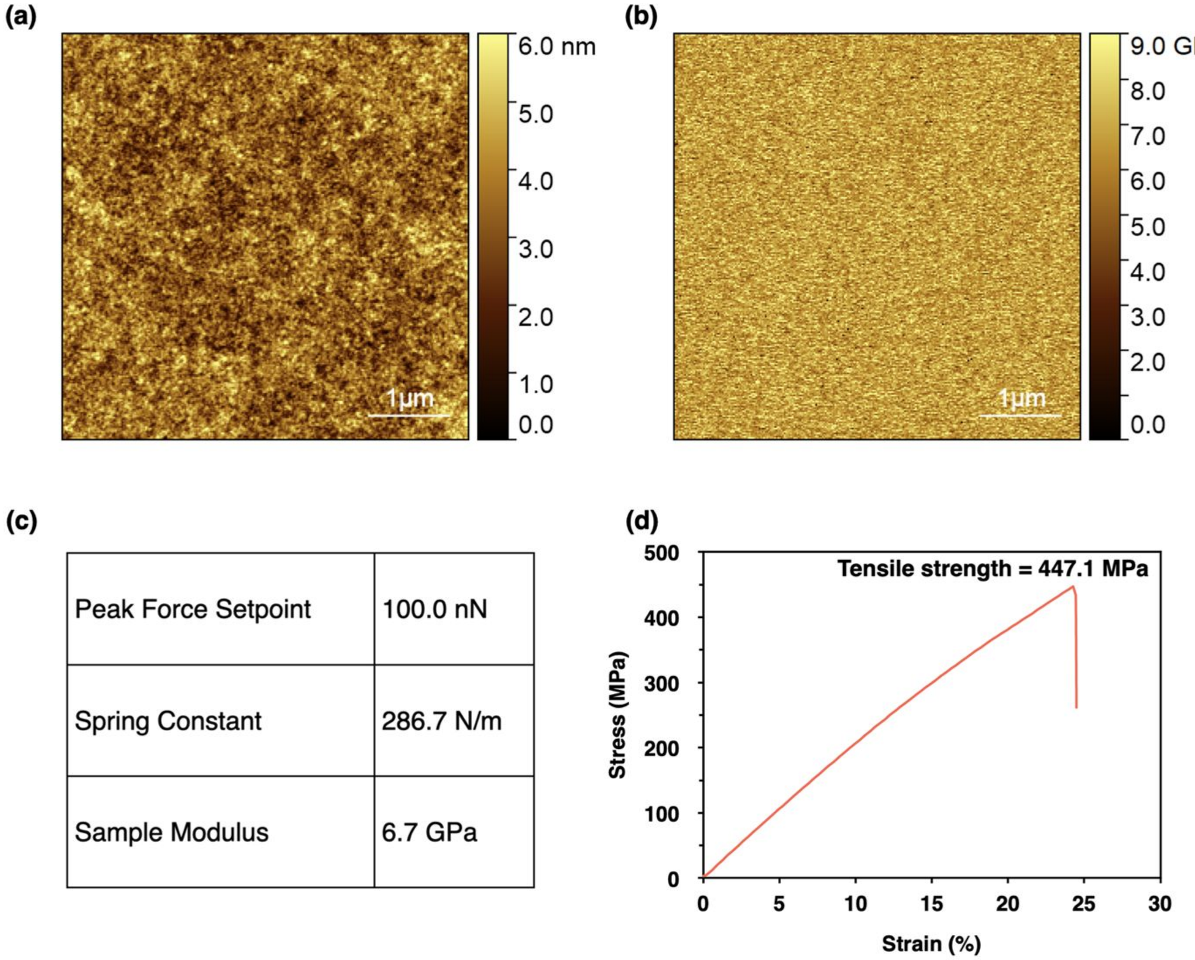

Figure S10. Measuring the mechanical strength of the TpPa-SO_3H membranes using AFM at a scanning range of 5 μm × 5 μm. (a) Height image. (b) DMT modulus image. (c) Measurement result. The average value of Young's modulus is 6.7 GPa for the selected area. (d) Stress-strain curve of TpPa-SO_3H membranes. The free-standing membrane displays high mechanical strength to withstand pressure-driven filtration.

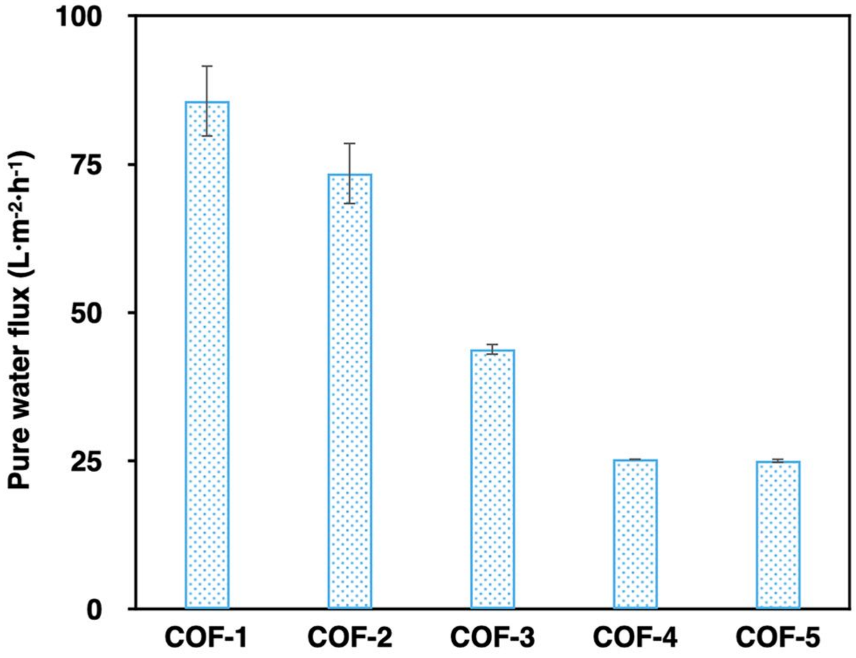

Figure S11. Pure water flux of Tp-PaSO_3H membranes with different thicknesses (0.2 MPa; 3.14 cm^{-2} effective membrane area; 25 ℃; each above value was based on the average of at least three independent samples).

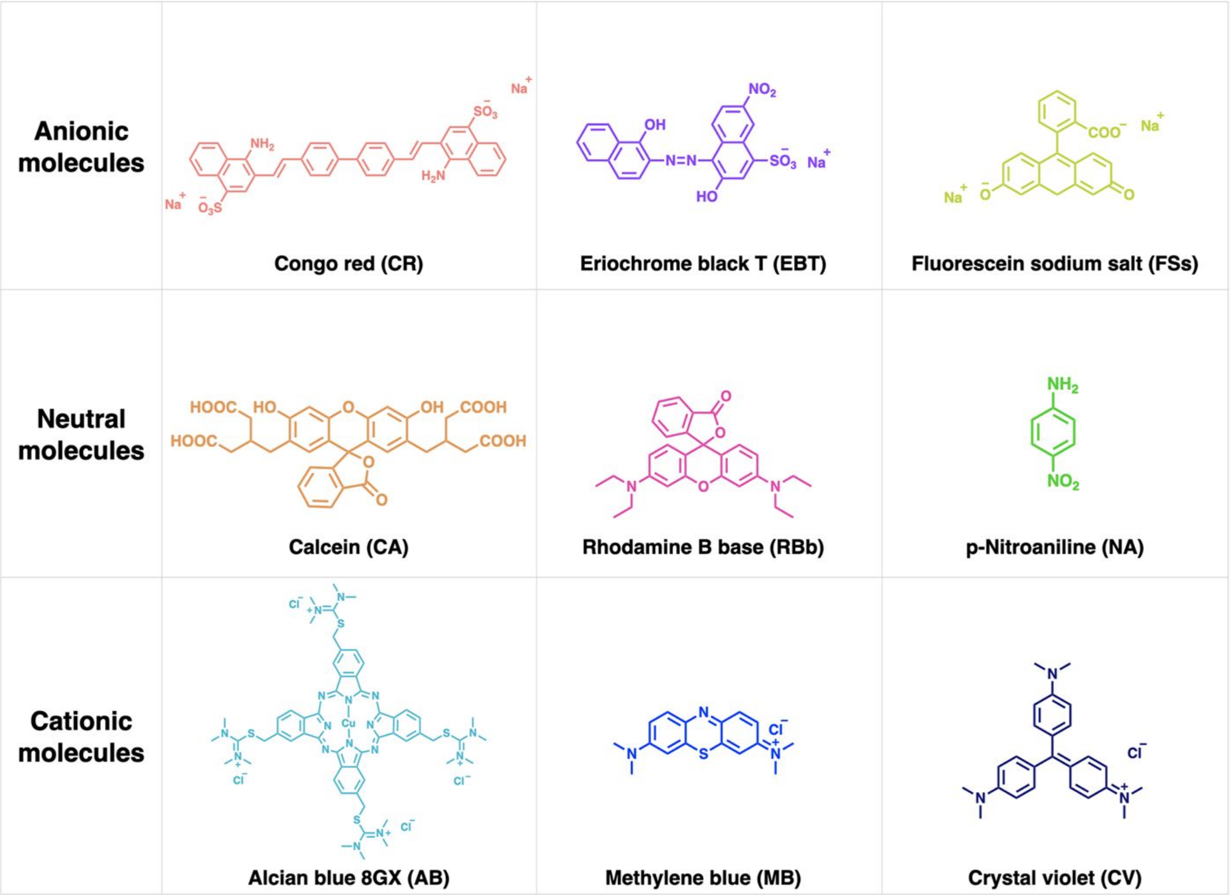

Figure S12. Chemical structures of different dye molecules are used in this work.

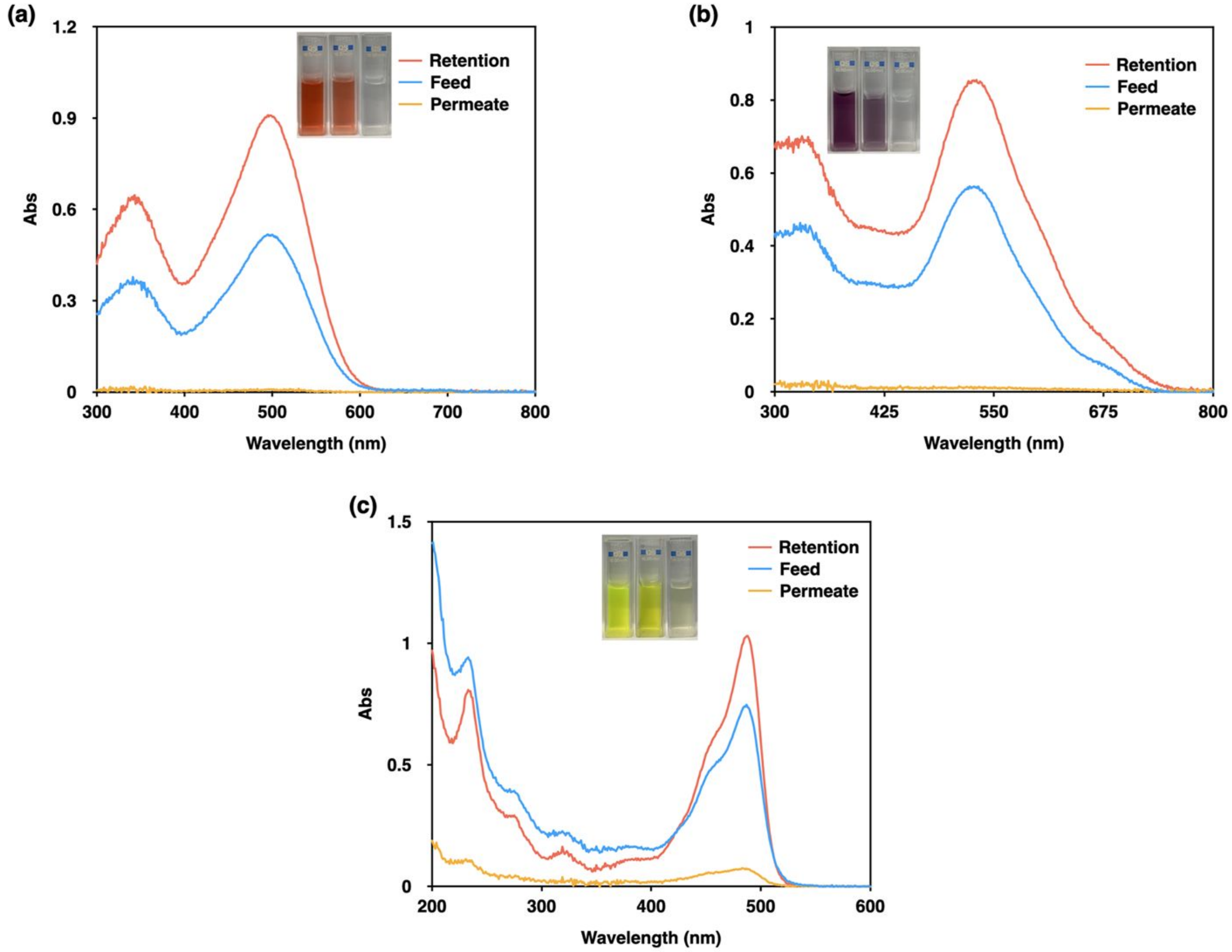

Figure S13. Concentration analysis result of (a) Congo red, (b) Eriochrome black T, (c) Fluorescein sodium salt by UV-vis spectroscopy of feed solution, permeate solution and retention solution.

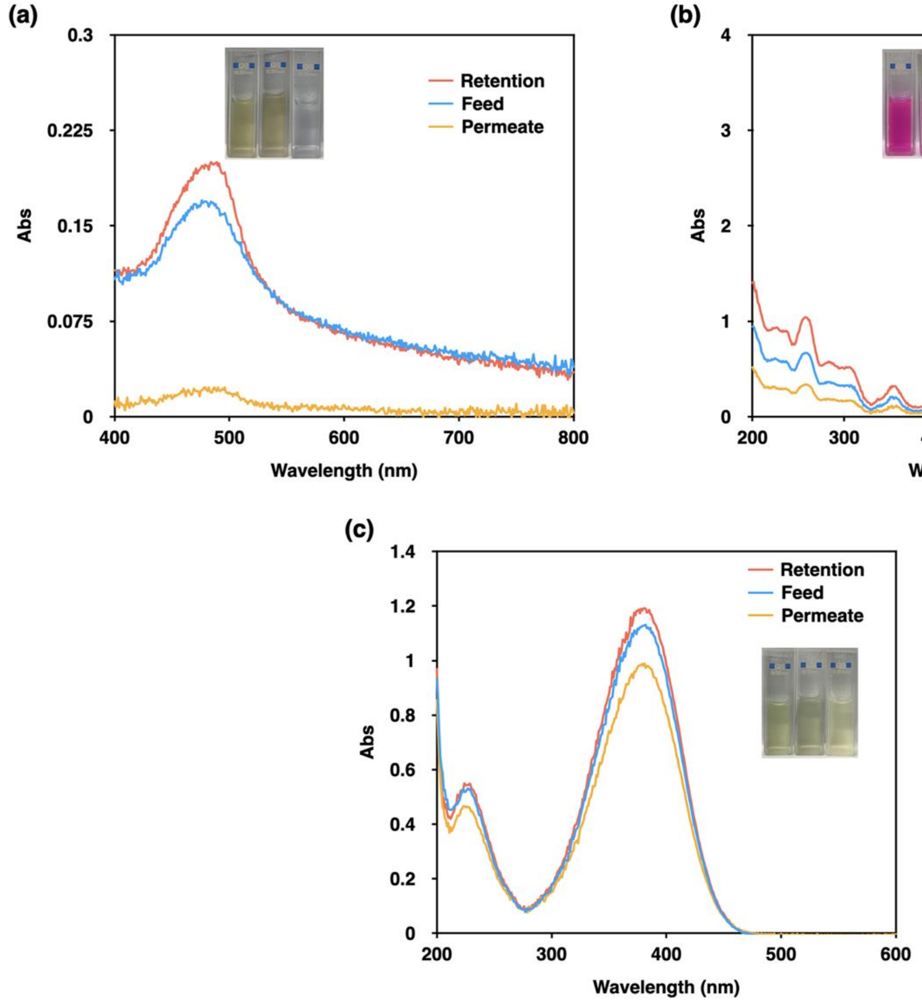

Figure S14. Concentration analysis result of (a) Calcein, (b) Rhodamine B base, (c) p-Nitroaniline by UV-vis spectroscopy of feed solution, permeate solution and retention solution.

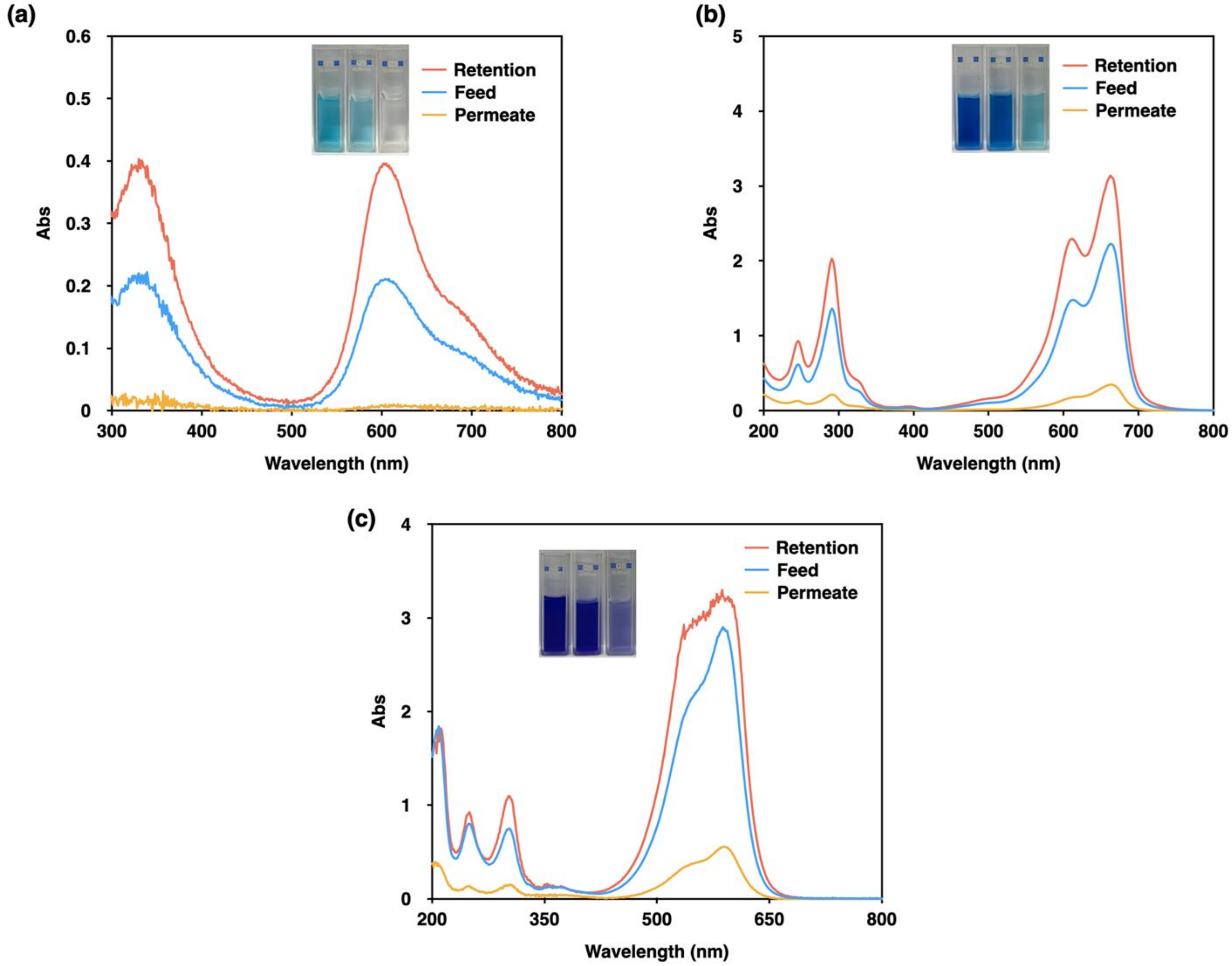

Figure S15. Concentration analysis result of (a) Alcian blue 8GX, (b) Methylene blue, (c) Crystal violet by UV-vis spectroscopy of feed solution, permeate solution and retention solution.

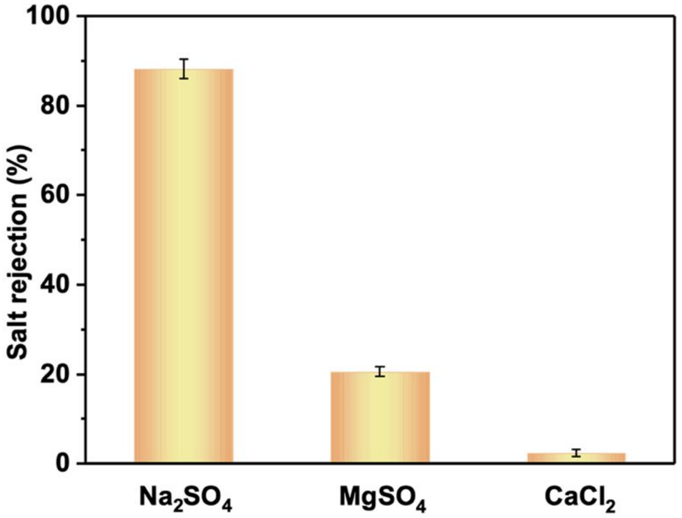

Figure S16. Salt rejection of TpPa-SO_3H membranes toward different divalent salts.

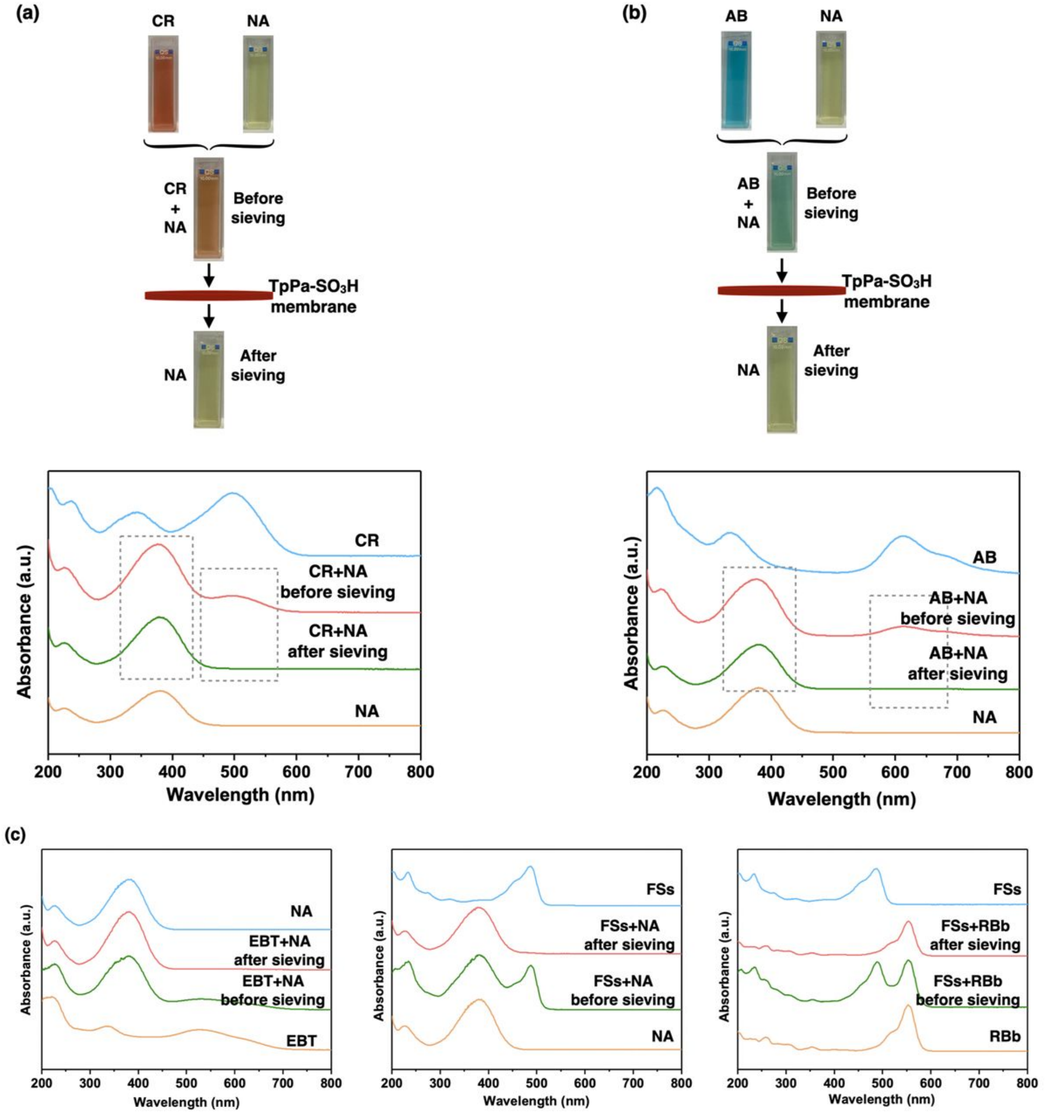

Figure S17. (a) Digital photos of sieving of CR from a mixture of CR and NA, and the corresponding UV spectra of the selective separation in the mixture of CR/NA. (b) Digital photos of sieving of AB from a mixture of AB and NA, and the corresponding UV spectra of the selective separation in the mixture of AB/NA (c) UV spectra of the selective separation in the mixture of EBT/NA, FSs/NA, and FSs/RBb.

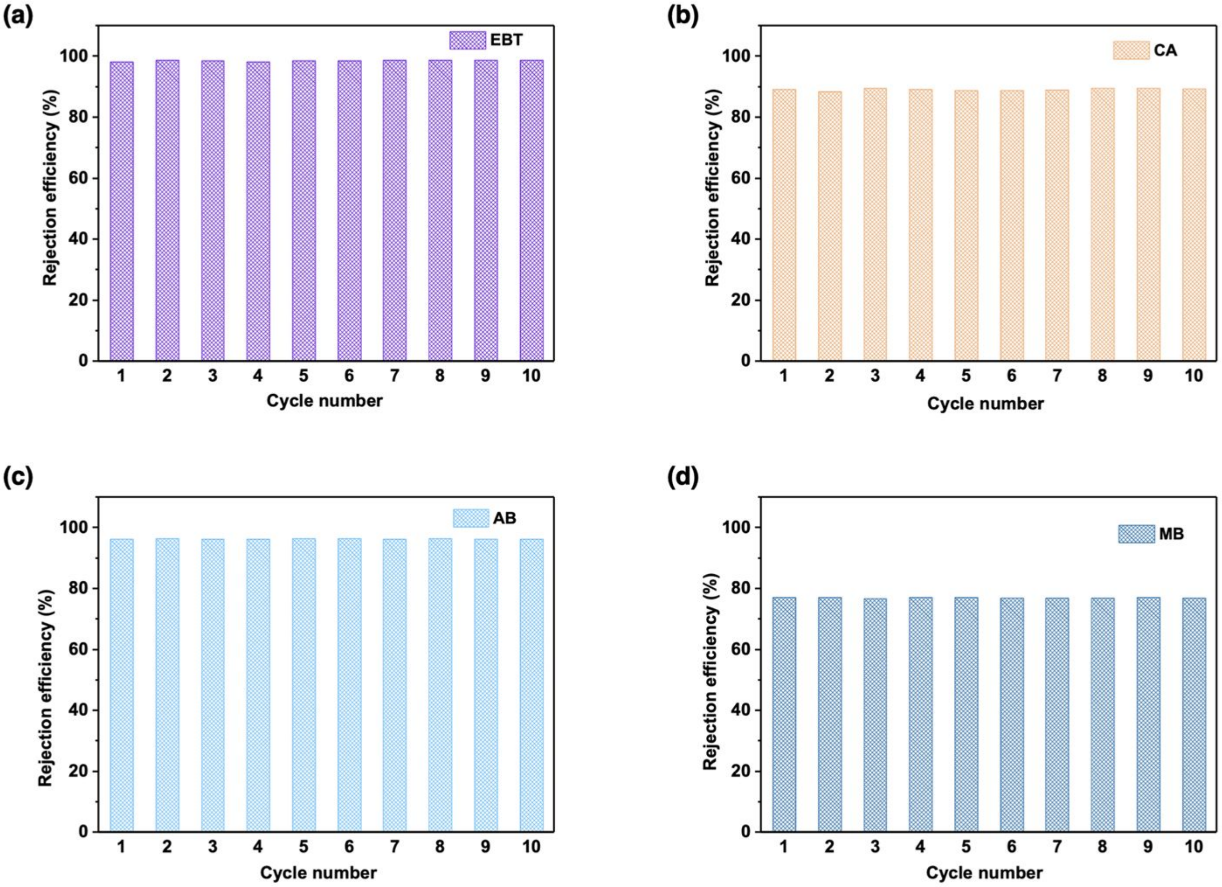

Figure S18. Rejection performance of TpPa-SO_3H membranes during the cycle experiments towards (a) EBT, (b) CA, (c) AB, and (d) MB dyes.

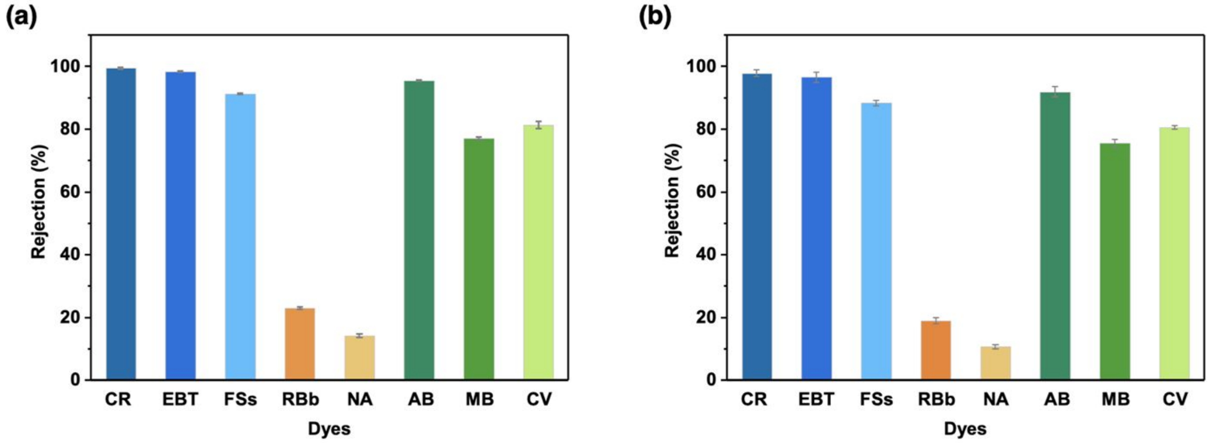

Figure S19. Rejection performance of TpPa-SO_3H membranes toward different concentrations of dyes in (a) 100 ppm and (b) 300 ppm, respectively.

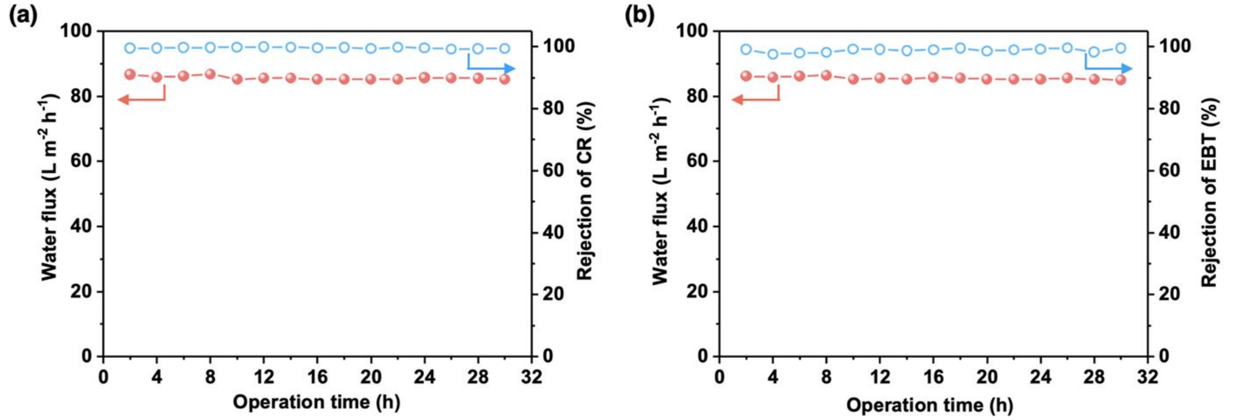

Figure S20. Long-term stability of the TpPa-SO_3H membrane towards (a) Congo red and (b) Eriochrome black T solution.

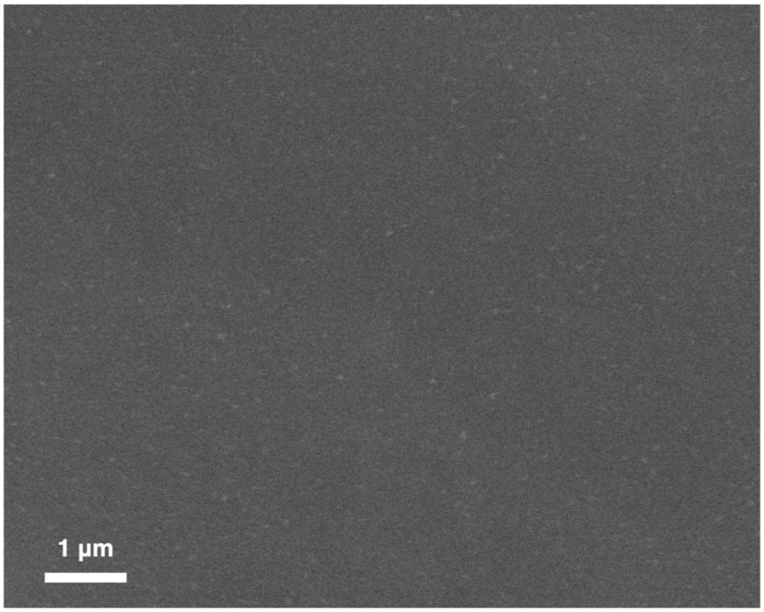

Figure S21. Surface SEM image of the membrane for long-term stability test after washing with DI water.

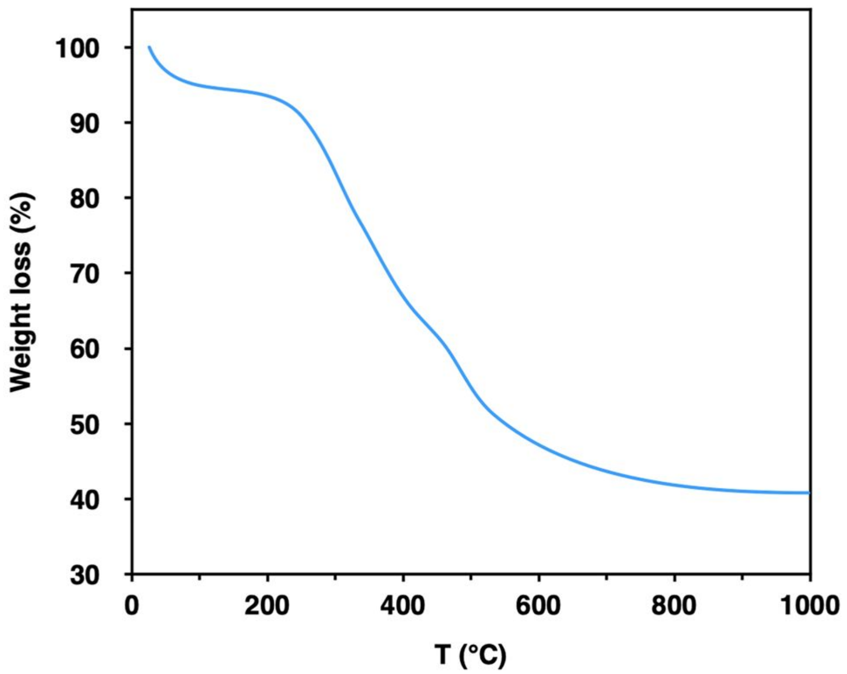

Figure S22. TGA of the TpPa-SO_3H COF membrane.

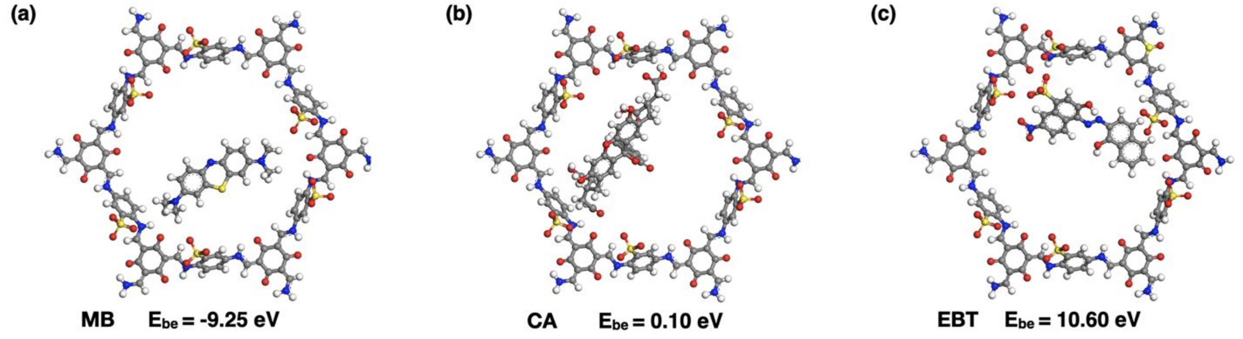

Figure S23. Simulated model and binding energy (E_{be}) of the complex consisting of dyes and TpPa-SO_3H membranes. (a) Membranes and MB composite. (b) Membranes and CA composite. (c) Membranes and EBT composite.

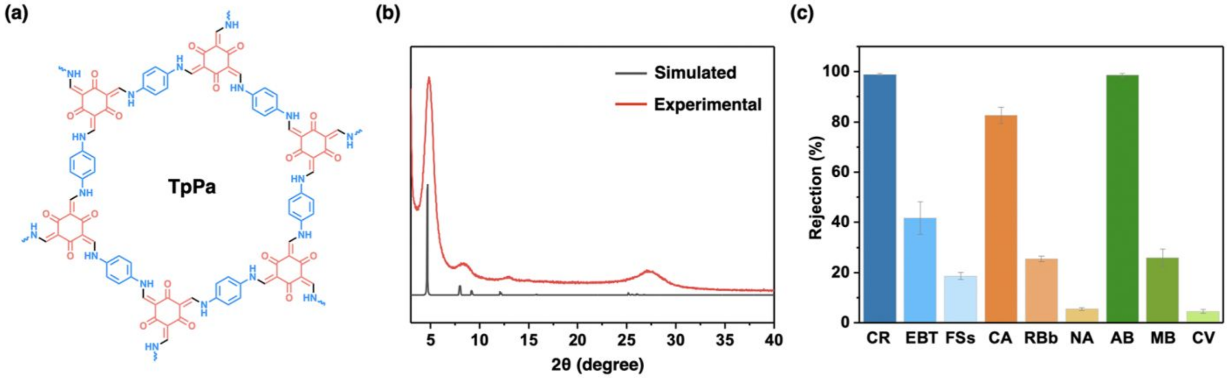

Figure S24. (a) Chemical structures of TpPa. (b) Comparison of the experimental PXRD patterns of TpPa with simulated eclipsed stacking model. (c) The rejection performance of the TpPa membrane for Congo red (CR), Eriochrome black T (EBT), Fluorescein sodium salt (FSs), Calcein (CA), Rhodamine B base (RBb), p-Nitroaniline (NA), Alcian blue 8GX (AB), Methylene blue (MB), and Crystal violet (CV) dyes. The result indicated that the rejection performance was influenced by the molecular size and shape. Only dyes with a molecular size exceeding the pore size were subjected to rejection.

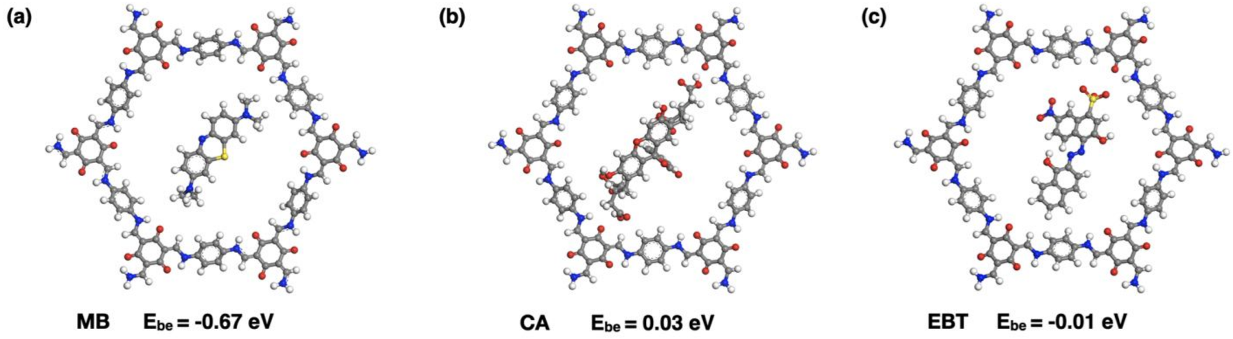

Figure S25. Simulated model and binding energy (E_{be}) of the complex consisting of dyes and TpPa membranes. (a) Membranes and MB composite. (b) Membranes and CA composite. (c) Membranes and EBT composite. The result revealed that the rejection performance is attributed to the size exclusion.

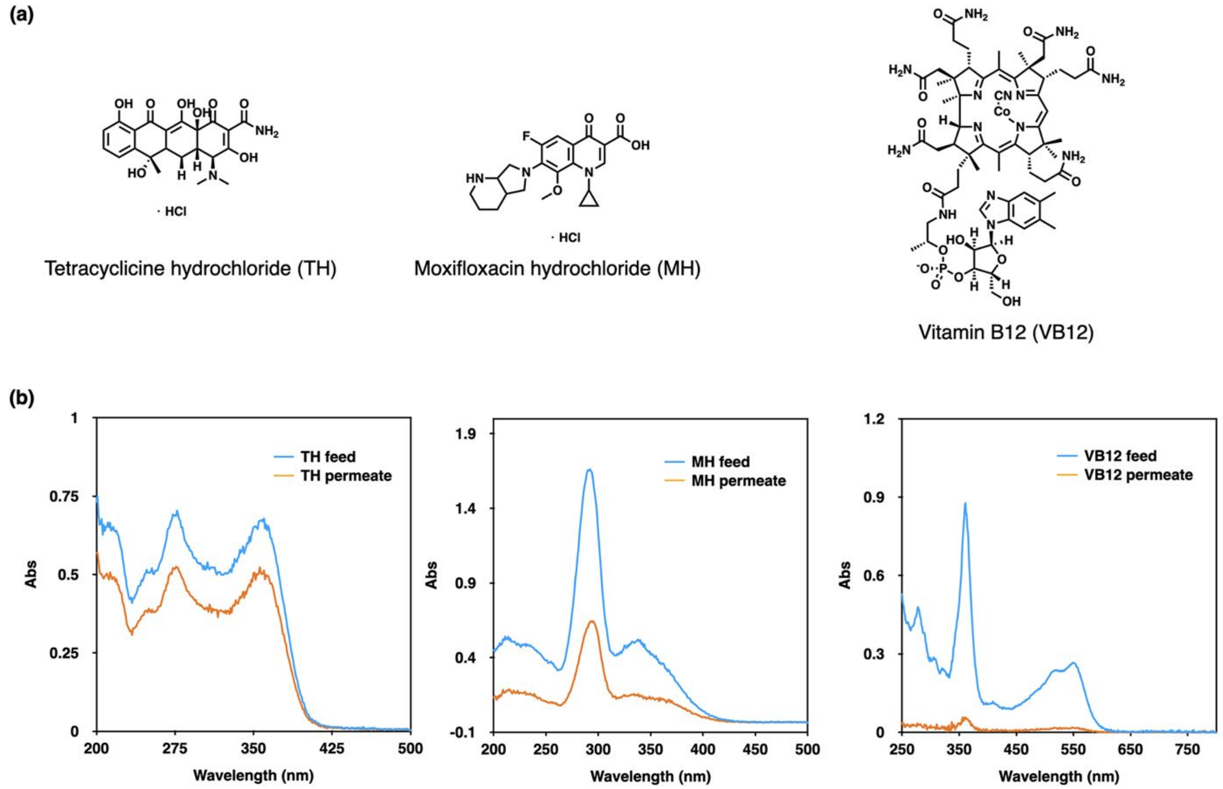

Figure S26. (a) Chemical structures of drug molecules. (b) Concentration analysis result of drugs by UV-vis spectroscopy of feed solution and permeate solution.

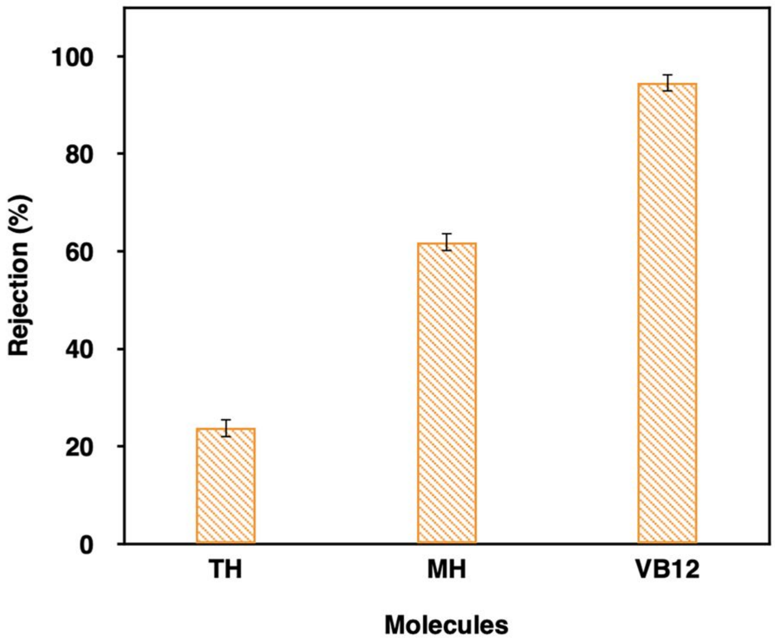

Figure S27. The rejection performance of the TpPa-SO_3H membrane for three drugs.

5. Tables.

Table S1. The concentration of monomers for the preparation of COF membrane with different thicknesses.

No.	Tp (mmol/L)	Pa-SO_3H (mmol/L)	Thickness (μm)
COF-1	9.83	14.73	0.6
COF-2	15.07	22.60	1.3
COF-3	19.67	29.50	3.7
COF-4	24.90	37.37	5.0
COF-5	29.97	44.97	8.7

Table S2. Atomic coordinates of the optimized AA-stacking structural model of TpPa-SO_3H (space group *P*3, a = 22.6269 Å; b = 22.6269 Å; c = 5.5280 Å, α = 90°; β = 90°; γ = 120°).

Atom	x/a	y/b	z/c
C1	0.47391	0.92686	-0.08024
C2	0.52489	0.96602	0.08825
C3	0.55035	0.03640	0.11099
C4	0.52487	0.06890	-0.04308
C5	0.47364	0.02901	-0.21105
C6	0.44880	0.95905	-0.23123
N7	0.44916	0.85484	-0.09753
N8	0.54769	0.14081	-0.03304
C9	0.61816	0.19229	-0.00744
C10	0.63969	0.25851	0.04282
C11	0.71380	0.30662	0.07332
O12	0.53387	0.24909	0.13715
C13	0.19283	0.57348	-0.17232
C14	0.25871	0.61813	-0.22600
C15	0.30745	0.59287	-0.25487
O16	0.24710	0.71361	-0.31158
S17	0.91992	0.53331	0.34669
O18	0.85699	0.46886	0.47715
O19	0.96811	0.57905	0.56619
O20	0.88631	0.57740	0.25249
H21	0.54426	0.94119	0.20531
H22	0.45359	0.05266	-0.33078
H23	0.41063	0.93042	-0.36722
H24	0.47822	0.83624	-0.01557
H25	0.51394	0.15552	-0.10082
H26	0.65593	0.17697	-0.03440
H27	0.17607	0.51948	-0.16578
H28	0.94884	0.59083	0.70120

Table S3. Performance comparison of various membranes towers dyes rejection.

Membrane type	Dye molecule	Rejection (%)	Permeance ($L\cdot m^{-2}\cdot h^{-1}\cdot bar^{-1}$)	Ref.
PVDF-SAN-60	Congo red	97.7	9.5	[S1]
	Reactive black 5	75	10.49	[S1]
Sepro NF 6	Congo red	99.93	13.3	[S2]
Sepro NF 2A	Congo red	99.96	9.585	[S2]
Ceramic NF	Chrome black T	>96.8	24.75	[S3]
PAA/PVA/GA	Congo red	96	4.2	[S4]
SiO2-PSS/PES	Congo red	99.8	25.225	[S5]
PEI-GO/PAA/PVA/GA	Methyl blue	99.3	0.87	[S6]
GO/MoS2	Methyl blue	97.4	10.2	[S7]
TA/GOQDs TFN	Methyl blue	98.1	11.665	[S8]
GO/PAN	Methyl orange	97.1	15.2	[S9]
GO NFM	Evans blue	98.68	20.23	[S10]
PPCNF@ZIF57.1	Methyl violet	98.3	23.7	[S11]
ZIF-8/PA	Congo red	99.98	2.26	[S12]
ZIF-8/PSS	Methyl blue	98.6	26.5	[S13]
ZIF-12/PAN	Methyl blue	99.4	27.2	[S14]
PEI/CMCNa/PP	Brilliant green	99.8	13.7	[S15]
	Crystal violet	97.9	13.4	[S15]
TFP-HHTTA	Crystal violet	>90	28.7	[S16]
TpPa/AAO	Acid fuchsin	93.5	14	[S17]
PSf/S-UiO-66	Methyl red	99.19	2.476	[S18]
Modified PEI/PAN	Methyl blue	97.3	25.5	[S19]
UiO-66-NH2@ZIF-8	Methyl orange	97	36.7	[S20]

SMWCNT/TFN	methylene blue	98.9	13.2	[S21]
G-CNTm	Methyl orange	>96	11.3	[S22]
Porphyrin/MPD	Brilliant blue	59	32.5	[S23]
TETA-TFN	Crystal violet	92	27.8	[S24]
□-CD-2.0	Methyl orange	91	5.5	[S25]
TpPa-SO_3H	Congo red	99.4	43	This work

Table S4. Characteristics and properties of the water-soluble dye molecules used for the membrane performance tests.

Dye molecule	Charge	M_w	λ_{max}	Molecular size
Congo red	Negative	696.66	504	2.56 nm x 0.73 nm
Eriochrome black T	Negative	461.38	530	1.55 nm x 0.88 nm
Fluorescein sodium salt	Negative	376.27	490	1.03 nm x 0.96 nm
Calcein	Neutral	622.53	490	1.76 nm x 0.88 nm
Rhodamine B base	Neutral	442.55	556	1.49 nm x 1.15 nm
p-Nitroaniline	Neutral	138.12	380	0.69 nm x 0.43 nm
Alcian blue 8GX	Postive	1298.86	615	2.22 nm x 2.08 nm
Methylene blue	Postive	319.85	665	1.52 nm x 0.75 nm
Crystal violet	Postive	407.99	590	0.91 nm x 0.91 nm

Table S5. Characteristics and properties of the water-soluble drug molecules used for the membrane performance test.

Molecule	M_w	λ_{max}	Molecular size
Tetracyclicine hydrochloride	480.9	356 nm	1.22 nm x 0.68 nm
Moxifloxacin hydrochloride	437.9	294 nm	1.42 nm x 0.86 nm
Vitamin B12	1355.39	361 nm	1.83 nm x 1.42 nm

6. Supplemental references.

[S1] Srivastava, H. P.; Arthanareeswaran, G.; Anantharaman, N.; Starov, V. M. Performance of modified poly(vinylidene fluoride) membrane for textile wastewater ultrafiltration. *Desalination* **2011**, *282*, 87-94.

[S2] Lin, J.; Ye, W.; Zeng, H.; Yang, H.; Shen, J.; Darvishmanesh, S.; Luis, P.; Sotto, A.; Bruggen, B. V. d. Fractionation of direct dyes and salts in aqueous solution using loose nanofiltration membranes. *J. Membr. Sci.* **2015**, *477*, 183-193.

[S3] Chen, P.; Ma, X.; Zhong, Z.; Zhang, F.; Xing, W.; Fan, Y. Performance of ceramic nanofiltration membrane for desalination of dye solutions containing NaCl and Na_2SO_4. *Desalination* **2017**, *404*, 102-111.

[S4] Wang, L.; Wang, N.; Zhang, G.; Ji, S. Covalent crosslinked assembly of tubular ceramic-based multilayer nanofiltration membranes for dye desalination. *AIChE Journal* **2013**, *59*, 3834-3842.

[S5] Xing, L.; Guo, N.; Zhang, Y.; Zhang, H.; Liu, J. A negatively charged loose nanofiltration membrane by blending with poly (sodium 4-styrene sulfonate) grafted SiO_2 via SI-ATRP for dye purification. *Sep. Purif. Technol.* **2015**, *146*, 50-59.

[S6] Wang, N.; Ji, S.; Zhang, G.; Li, J.; Wang, L. Self-assembly of graphene oxide and polyelectrolyte complex nanohybrid membranes for nanofiltration and pervaporation. *Chem. Eng. J.*, **2012**, *213*, 318-329.

[S7] Zhang, P.; Gong, J.-L.; Zeng, G.-M.; Song, B.; Cao, W., Liu, H.-Y.; Huan, S.-Y.; Peng, P. Novel “loose” GO/MoS_2 composites membranes with enhanced permeability for effective salts and dyes rejection at low pressure. *J. Membr. Sci.*, **2019**, *574*, 112-123.

[S8] Zhang, C.; Wei, K.; Zhang, W.; Bai, Y.; Sun, Y.; Gu, J. Graphene oxide quantum dots incorporated into a thin film nanocomposite membrane with high flux and antifouling properties for low-pressure nanofiltration. *ACS Appl. Mater. Interfaces* **2017**, *9*, 11082-11094.

[S9] Zhang, M.; Sun, J.; Mao, Y.; Liu, G.; Jin, W. Effect of substrate on formation and nanofiltration performance of graphene oxide membranes. *J. Membr. Sci.*, **2019**, *574*, 196-204.

[S10] Chen, L.; Moon, J.-H.; Ma, X.; Zhang, L.; Chen, Q.; Chen, L.; Peng, R.; Si, P.; Feng, J.; Li, Y.; Lou, J.; Ci, L. High performance graphene oxide nanofiltration membrane prepared by electrospraying for wastewater purification. *Carbon* **2018**, *130*, 487-494.

[S11] Zhang, W.; Yang, K.; Han, X.; Cai, H.; Lu, W.; Yuan, Y.; Zhang, S.; Gao, F. Metal-organic frameworks decorated pomelo peel cellulose nanofibers membranes for high performance dye rejection. *Colloids and Surfaces A: Physicochemical and Engineering Aspects* **2022**, *649*, 129393.
[S12] Wang, L.; Fang, M.; Liu, J.; He, J.; Deng, L.; Li, J.; Lei, J. The influence of dispersed phases on polyamide/ ZIF-8 nanofiltration membranes for dye removal from water. *RSC Adv.* **2015**, *5*, 50942-50954.
[S13] Zhang, R.; Ji, S.; Wang, N.; Wang, L.; Zhang G.; Li, J.-R. Coordination-driven in situ self-assembly strategy for the preparation of metal–organic framework hybrid membranes. *Angew. Chem. Int. Ed.* **2014**, *53*, 9775-9779.
[S14] Wang, N.; Li, X.; Wang, L.; Zhang, L.; Zhang G.; Ji, S. Nanoconfined zeolitic imidazolate framework membranes with composite layers of nearly zero thickness. *ACS Appl. Mater. Interfaces* **2016**, *8*, 21979-21983.
[S15] Chen, Q.; Yu, P.; Huang, W.; Yu, S.; Liu, M.; Gao, C. High-flux composite hollow fiber nanofiltration membranes fabricated through layer-by-layer deposition of oppositely charged crosslinked polyelectrolytes for dye removal. *J. Membr. Sci.* **2015**, *492*, 312-321.
[S16] Shinde, D. B.; Cao, L.; Liu, X.; Wonanke, D. A.D.; Zhou, Z.; Hedhili, M. N.; Addicoat, M.; Huang, K. -W.; Lai, Z. Tailored pore size and microporosity of covalent organic framework (COF) membranes for improved molecular separation. *J. Membr. Sci. Lett.* **2021**, *1*, 100008.
[S17] Shi, X.; Xiao, A.; Zhang, C.; Wang, Y. Growing covalent organic frameworks on porous substrates for molecule-sieving membranes with pores tunable from ultra- to nanofiltration. *J. Membr. Sci.* **2019**, *576*, 116-122.
[S18] Ahmadipouya, S.; Mousavi, S. A.; Shokrgozar, A.; Mousavi, D. V. Improving dye removal and antifouling performance of polysulfone nanofiltration membranes by incorporation of UiO-66 metal-organic framework. *J. Environ. Chem. Eng.* **2022**, *10*, 107535.
[S19] Zhao S., Wang, Z. A loose nano-filtration membrane prepared by coating HPAN UF membrane with modified PEI for dye reuse and desalination. *J. Membr. Sci.* **2017**, *524*, 214-224.
[S20] Xu, Y.; Zhao, X.; Chang, R.; Qu, H.; Xu, J.; Ma, J. Designing heterogeneous MOF-on-MOF membrane with hierarchical pores for effective water treatment. *J. Membr. Sci.* **2022**, *658*, 120737.
[S21] Zheng, J.; Li, M.; Yu, K.; Hu, J.; Zhang, X.; Wang, L. Sulfonated multiwall carbon nanotubes assisted thin-film nanocomposite membrane with enhanced water flux and anti-fouling property. *J. Membr. Sci.* **2017**, *524*, 344-353.

[S22] Han, Y.; Jiang Y.; Gao, C. High-flux graphene oxide nanofiltration membrane intercalated by carbon nanotubes. *ACS Appl. Mater. Interfaces* **2015**, *7*, 8147-8155.

[S23] Duong, P. H.H.; Anjum, D. H.; Peinemann, K. -V.; Nunes, S. P. Thin porphyrin composite membranes with enhanced organic solvent transport. *J. Membr. Sci.* **2018**, *563*, 684-693.

[S24] Soroko, I.; Livingston, A. Impact of TiO_2 nanoparticles on morphology and performance of crosslinked polyimide organic solvent nanofiltration (OSN) membranes. *J. Membr. Sci.*, **2009**, *343*, 189-198.

[S25] Villalobos, L. F.; Huang, T.; Peinemann, K. -V. Cyclodextrin films with fast solvent transport and shape-selective permeability. *Adv. Mater.* **2017**, *29*, 1606641.
